# Supplementary material for: The impact of positive surgical margin parameters and pathological stage on biochemical recurrence after radical prostatectomy: A systematic review and meta-analysis
Source: PLoS One. 2024 Jul 11;19(7):e0301653. doi: 10.1371/journal.pone.0301653 (PMC11239040; doi:10.1371/journal.pone.0301653)
Supplement: S1 Table — (PDF) [file pone.0301653.s001.pdf]

Supplementary Table 1. NOS Scores of included studies.

Study	Representativeness of the exposed cohort★	Selection of the non-exposed cohort★	Ascertainment of exposure★	Demonstration that outcome of interest was not present at start of the study★	Comparability of cohorts on the basis of the design or analysis★★	Assessment of outcome★	Was follow-up long enough for outcomes to occur★	Adequacy of follow up of cohorts★	Total score s
Sasaki 2023	★	★	★	★	★	★	★	★	8
Preisser 2019	★	★	★	★	★	★	★	★	8
Viers 2014	★	★	★	★	★	★	★	★	8
Chapin 2017	★	★	★	★	★	★	★	★	7
Savdie 2011	★	★	★	★		★	★	★	7
Huang 2013	★	★	★	★	★	★		★	7
Lee 2020	★	★	★	★		★	★	★	7
Dason 2021	★	★	★	★		★	★	★	7
Ginzburg 2012	★	★	★	★	★	★		★	7
Kozal 2015	★	★	★	★	★	★		★	7
Ploussard 2014	★	★	★	★	★	★		★	7
Dev 2015	★	★	★	★	★	★		★	7
Kordan 2009	★	★	★	★	★	★		★	7
Godoy 2009	★	★	★	★	★	★		★	7
Resnick 2010	★	★	★	★	★	★	★	★	8
Porpiglia 2011	★	★	★	★	★	★	★	★	8
Somford 2011	★	★	★	★	★	★		★	6
Lee 2013	★	★	★	★	★	★		★	7
Song 2017	★	★	★	★	★	★		★	7

Wu 2018	★	★	★	★	★	★	★	★	8
Simon 2006	★	★	★	★	★	★		★	7
Stephenson 2009	★	★	★	★	★	★		★	7
May 2011	★	★	★	★	★	★		★	7
Lian 2020	★	★	★	★	★	★		★	6
Oort 2010	★	★	★	★		★		★	6
Richters 2015	★	★		★	★	★	★	★	7
Keller 2018	★	★	★	★	★	★		★	7
Karl 2015	★	★		★	★	★	★	★	7
Iremashvili 2018	★	★		★	★	★	★	★	7
Porcaro 2019	★	★	★	★	★	★		★	7
Sooriakumaran 2015	★	★	★	★	★	★		★	7
