# Supplementary figures and images for: The impact of positive surgical margin parameters and pathological stage on biochemical recurrence after radical prostatectomy: A systematic review and meta-analysis
Source: PLoS One. 2024 Jul 11;19(7):e0301653. doi: 10.1371/journal.pone.0301653 (PMC11239040; doi:10.1371/journal.pone.0301653)

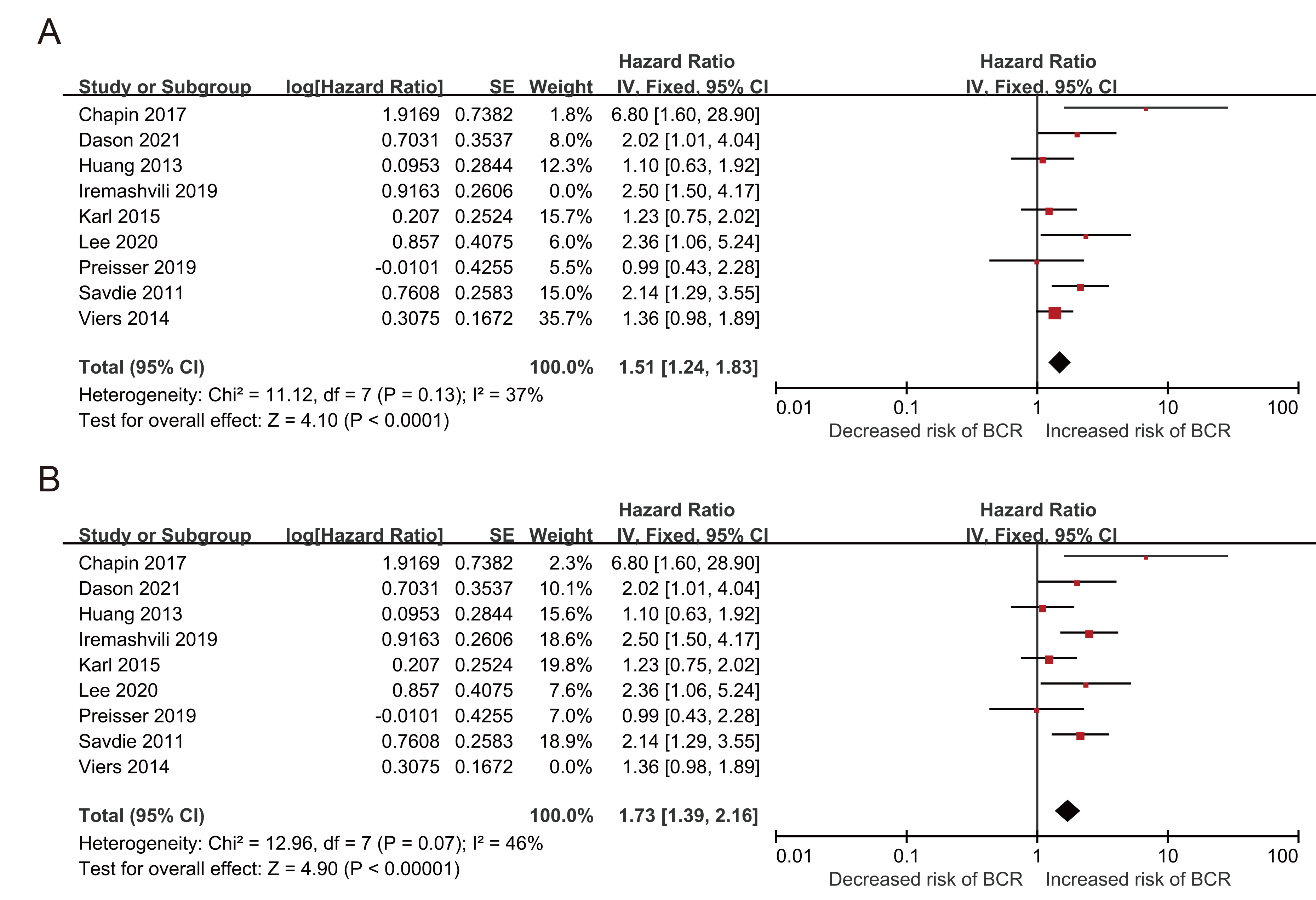

Supplement: S1 Fig — (A) Forest plots of studies excluded Iremashvili’s study evaluating the association between PGG and BCR, (B) Forest plots of studies excluded Vier’s study evaluating the association between PGG and BCR. (TIF) [file pone.0301653.s002.tif]

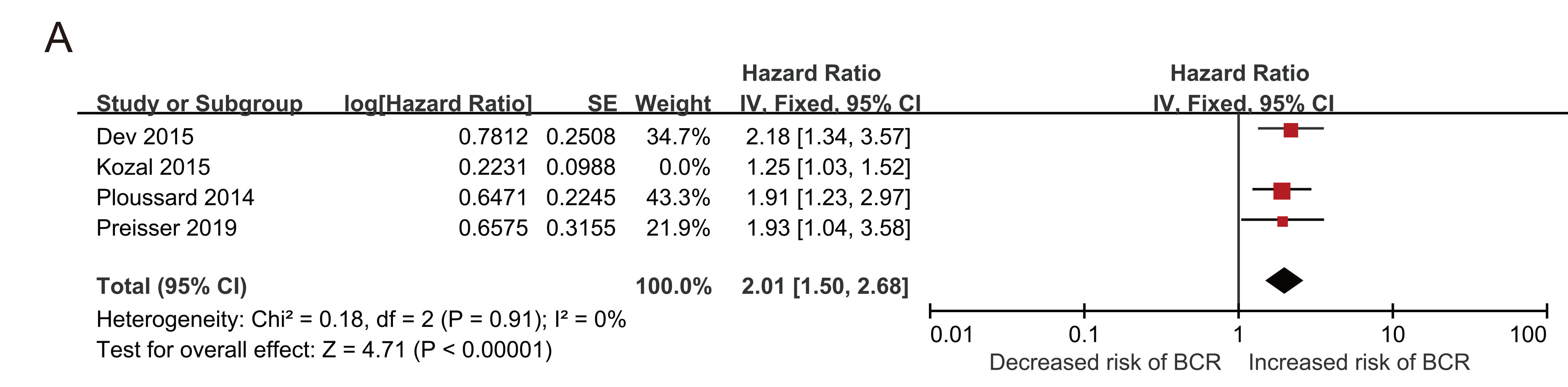

Supplement: S2 Fig — (TIF) [file pone.0301653.s003.tif]

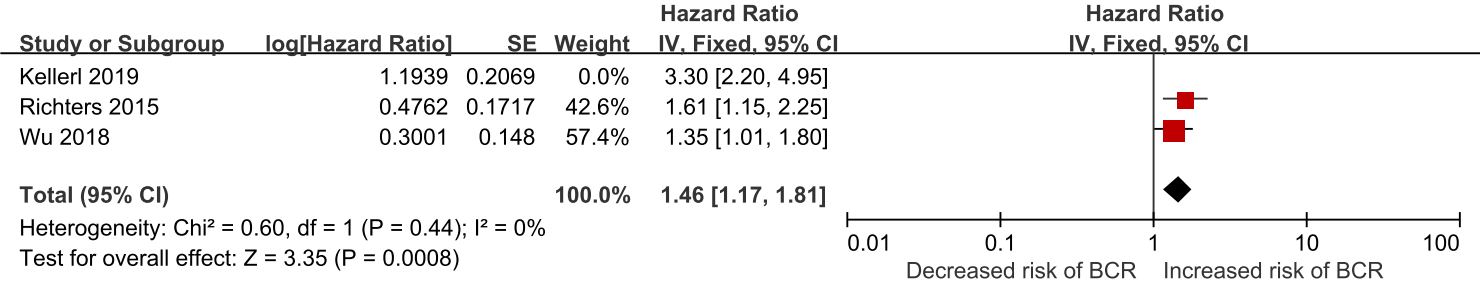

Supplement: S5 File — (PDF) [file pone.0301653.s008.pdf]
