# Supplementary material for: The impact of positive surgical margin parameters and pathological stage on biochemical recurrence after radical prostatectomy: A systematic review and meta-analysis
Source: PLoS One. 2024 Jul 11;19(7):e0301653. doi: 10.1371/journal.pone.0301653 (PMC11239040; doi:10.1371/journal.pone.0301653)

1. Forest plots of studies excluded Chapin's study evaluating the association between PGG and BCR

Study or Subgroup	log[Hazard Ratio]	SE	Weight	Hazard Ratio IV, Fixed, 95% CI
Chapin 2017	1.9169	0.7382	0.0%	6.80 [1.60, 28.90]
Dason 2021	0.7031	0.3537	7.1%	2.02 [1.01, 4.04]
Huang 2013	0.0953	0.2844	10.9%	1.10 [0.63, 1.92]
Iremashvili 2019	0.9163	0.2606	13.0%	2.50 [1.50, 4.17]
Karl 2015	0.207	0.2524	13.9%	1.23 [0.75, 2.02]
Lee 2020	0.857	0.4075	5.3%	2.36 [1.06, 5.24]
Preisser 2019	-0.0101	0.4255	4.9%	0.99 [0.43, 2.28]
Savdie 2011	0.7608	0.2583	13.3%	2.14 [1.29, 3.55]
Viers 2014	0.3075	0.1672	31.6%	1.36 [0.98, 1.89]
**Total (95% CI)**			**100.0%**	**1.57 [1.31, 1.89]**

Heterogeneity: $Chi^2 = 10.54$, df = 7 (P = 0.16); $I^2 = 34\%$

Test for overall effect: Z = 4.80 (P < 0.00001)

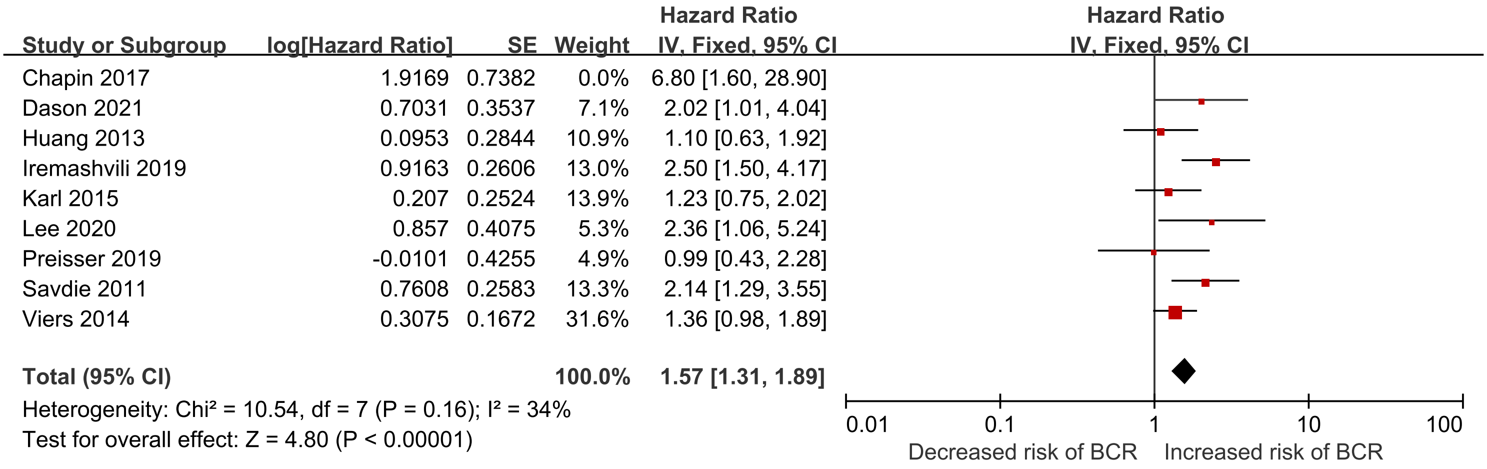

2. Forest plots of studies excluded Dason's study evaluating the association between PGG and BCR

Study or Subgroup	log[Hazard Ratio]	SE	Weight	Hazard Ratio IV, Fixed, 95% CI
Chapin 2017	1.9169	0.7382	1.7%	6.80 [1.60, 28.90]
Dason 2021	0.7031	0.3537	0.0%	2.02 [1.01, 4.04]
Huang 2013	0.0953	0.2844	11.6%	1.10 [0.63, 1.92]
Iremashvili 2019	0.9163	0.2606	13.8%	2.50 [1.50, 4.17]
Karl 2015	0.207	0.2524	14.7%	1.23 [0.75, 2.02]
Lee 2020	0.857	0.4075	5.6%	2.36 [1.06, 5.24]
Preisser 2019	-0.0101	0.4255	5.2%	0.99 [0.43, 2.28]
Savdie 2011	0.7608	0.2583	14.0%	2.14 [1.29, 3.55]
Viers 2014	0.3075	0.1672	33.5%	1.36 [0.98, 1.89]
**Total (95% CI)**			**100.0%**	**1.58 [1.31, 1.91]**

Heterogeneity: Chi² = 13.97, df = 7 (P = 0.05); I² = 50%

Test for overall effect: Z = 4.73 (P < 0.00001)

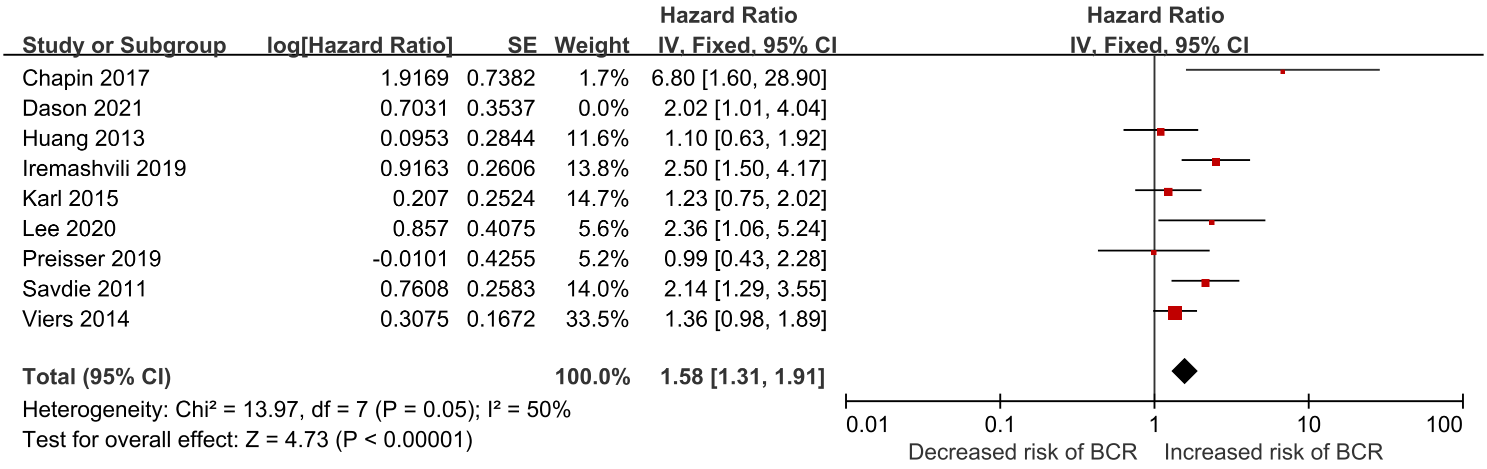

3. Forest plots of studies excluded Huang's study evaluating the association between PGG and BCR

Study or Subgroup	log[Hazard Ratio]	SE	Weight	Hazard Ratio IV, Fixed, 95% CI
Chapin 2017	1.9169	0.7382	1.8%	6.80 [1.60, 28.90]
Dason 2021	0.7031	0.3537	7.8%	2.02 [1.01, 4.04]
Huang 2013	0.0953	0.2844	0.0%	1.10 [0.63, 1.92]
Iremashvili 2019	0.9163	0.2606	14.4%	2.50 [1.50, 4.17]
Karl 2015	0.207	0.2524	15.3%	1.23 [0.75, 2.02]
Lee 2020	0.857	0.4075	5.9%	2.36 [1.06, 5.24]
Preisser 2019	-0.0101	0.4255	5.4%	0.99 [0.43, 2.28]
Savdie 2011	0.7608	0.2583	14.6%	2.14 [1.29, 3.55]
Viers 2014	0.3075	0.1672	34.9%	1.36 [0.98, 1.89]
**Total (95% CI)**			**100.0%**	**1.68 [1.39, 2.04]**

Heterogeneity: Chi² = 12.42, df = 7 (P = 0.09); I² = 44%
Test for overall effect: Z = 5.27 (P < 0.00001)

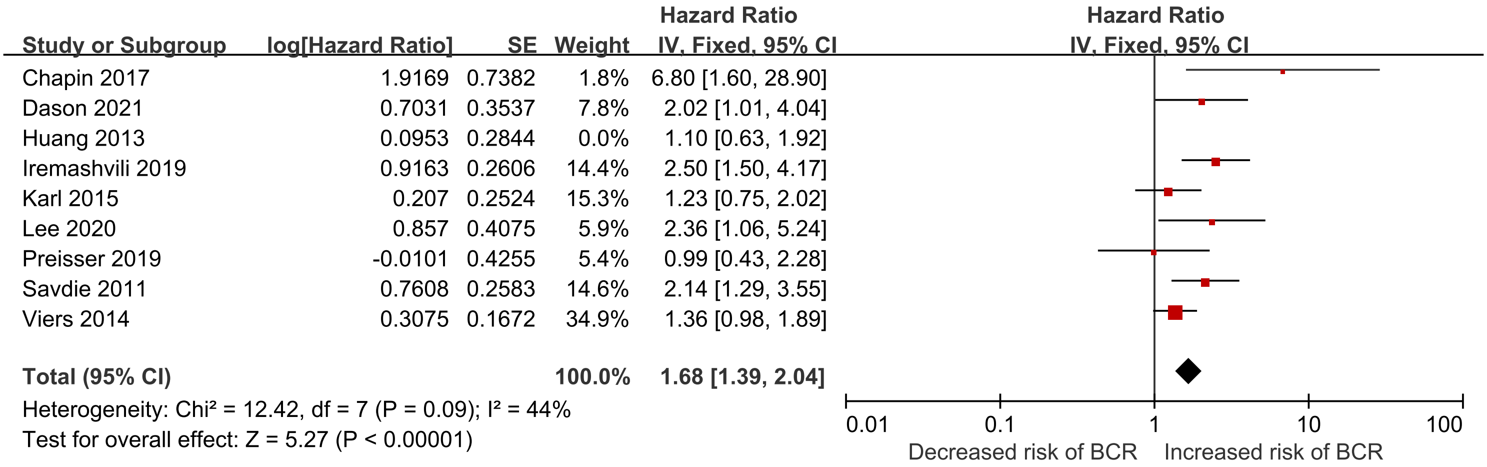

4. Forest plots of studies excluded Iremashvili's study evaluating the association between PGG and BCR

Study or Subgroup	log[Hazard Ratio]	SE	Weight	Hazard Ratio IV, Fixed, 95% CI
Chapin 2017	1.9169	0.7382	1.8%	6.80 [1.60, 28.90]
Dason 2021	0.7031	0.3537	8.0%	2.02 [1.01, 4.04]
Huang 2013	0.0953	0.2844	12.3%	1.10 [0.63, 1.92]
Iremashvili 2019	0.9163	0.2606	0.0%	2.50 [1.50, 4.17]
Karl 2015	0.207	0.2524	15.7%	1.23 [0.75, 2.02]
Lee 2020	0.857	0.4075	6.0%	2.36 [1.06, 5.24]
Preisser 2019	-0.0101	0.4255	5.5%	0.99 [0.43, 2.28]
Savdie 2011	0.7608	0.2583	15.0%	2.14 [1.29, 3.55]
Viers 2014	0.3075	0.1672	35.7%	1.36 [0.98, 1.89]
**Total (95% CI)**			**100.0%**	**1.51 [1.24, 1.83]**

Heterogeneity: $Chi^2 = 11.12$, $df = 7$ ($P = 0.13$); $I^2 = 37\%$

Test for overall effect: $Z = 4.10$ ($P < 0.0001$)

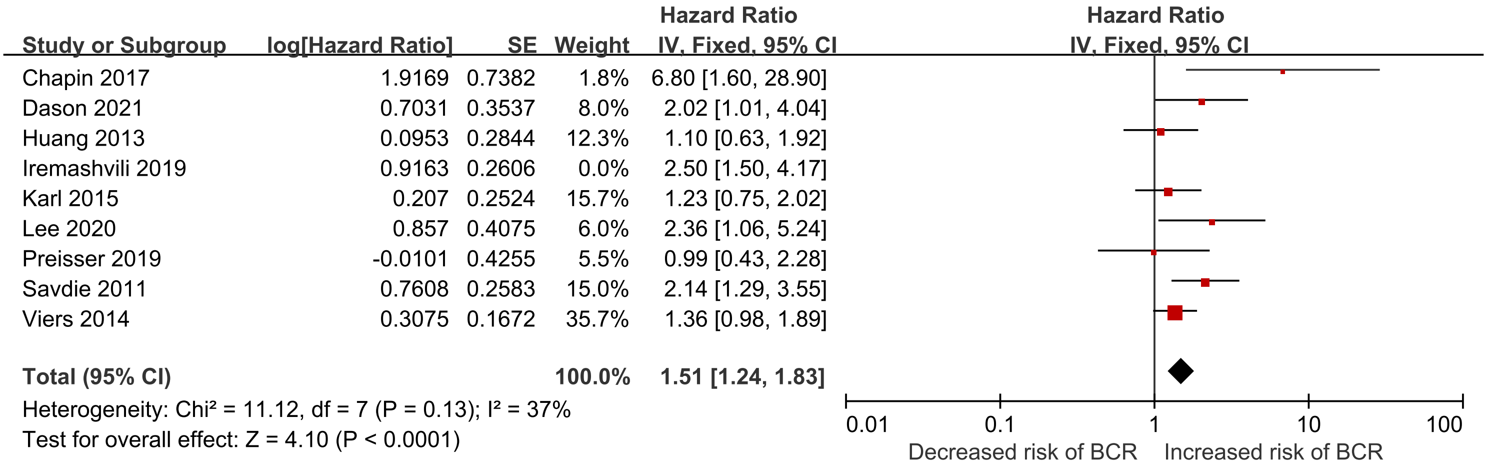

5. Forest plots of studies excluded Karl's study evaluating the association between PGG and BCR

Study or Subgroup	log[Hazard Ratio]	SE	Weight	Hazard Ratio IV, Fixed, 95% CI
Chapin 2017	1.9169	0.7382	1.8%	6.80 [1.60, 28.90]
Dason 2021	0.7031	0.3537	8.1%	2.02 [1.01, 4.04]
Huang 2013	0.0953	0.2844	12.5%	1.10 [0.63, 1.92]
Iremashvili 2019	0.9163	0.2606	14.8%	2.50 [1.50, 4.17]
Karl 2015	0.207	0.2524	0.0%	1.23 [0.75, 2.02]
Lee 2020	0.857	0.4075	6.1%	2.36 [1.06, 5.24]
Preisser 2019	-0.0101	0.4255	5.6%	0.99 [0.43, 2.28]
Savdie 2011	0.7608	0.2583	15.1%	2.14 [1.29, 3.55]
Viers 2014	0.3075	0.1672	36.1%	1.36 [0.98, 1.89]
**Total (95% CI)**			**100.0%**	**1.68 [1.38, 2.04]**

Heterogeneity: Chi² = 13.11, df = 7 (P = 0.07); I² = 47%

Test for overall effect: Z = 5.15 (P < 0.00001)

Hazard Ratio
IV, Fixed, 95% CI

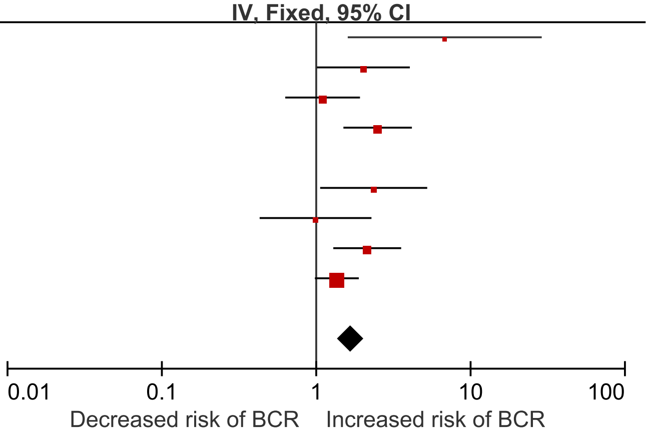

6. Forest plots of studies excluded Lee's study evaluating the association between PGG and BCR

Study or Subgroup	log[Hazard Ratio]	SE	Weight	Hazard Ratio IV, Fixed, 95% CI
Chapin 2017	1.9169	0.7382	1.7%	6.80 [1.60, 28.90]
Dason 2021	0.7031	0.3537	7.3%	2.02 [1.01, 4.04]
Huang 2013	0.0953	0.2844	11.4%	1.10 [0.63, 1.92]
Iremashvili 2019	0.9163	0.2606	13.5%	2.50 [1.50, 4.17]
Karl 2015	0.207	0.2524	14.4%	1.23 [0.75, 2.02]
Lee 2020	0.857	0.4075	0.0%	2.36 [1.06, 5.24]
Preisser 2019	-0.0101	0.4255	5.1%	0.99 [0.43, 2.28]
Savdie 2011	0.7608	0.2583	13.8%	2.14 [1.29, 3.55]
Viers 2014	0.3075	0.1672	32.8%	1.36 [0.98, 1.89]
**Total (95% CI)**			**100.0%**	**1.57 [1.30, 1.90]**

Heterogeneity: Chi² = 13.49, df = 7 (P = 0.06); I² = 48%

Test for overall effect: Z = 4.73 (P < 0.00001)

Hazard Ratio
IV, Fixed, 95% CI

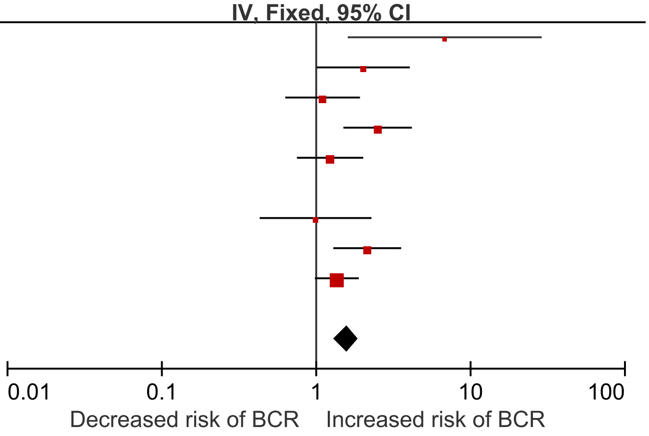

7. Forest plots of studies excluded Preisser's study evaluating the association between PGG and BCR

Study or Subgroup	log[Hazard Ratio]	SE	Weight	Hazard Ratio IV, Fixed, 95% CI
Chapin 2017	1.9169	0.7382	1.7%	6.80 [1.60, 28.90]
Dason 2021	0.7031	0.3537	7.3%	2.02 [1.01, 4.04]
Huang 2013	0.0953	0.2844	11.3%	1.10 [0.63, 1.92]
Iremashvili 2019	0.9163	0.2606	13.5%	2.50 [1.50, 4.17]
Karl 2015	0.207	0.2524	14.3%	1.23 [0.75, 2.02]
Lee 2020	0.857	0.4075	5.5%	2.36 [1.06, 5.24]
Preisser 2019	-0.0101	0.4255	0.0%	0.99 [0.43, 2.28]
Savdie 2011	0.7608	0.2583	13.7%	2.14 [1.29, 3.55]
Viers 2014	0.3075	0.1672	32.7%	1.36 [0.98, 1.89]
**Total (95% CI)**			**100.0%**	**1.65 [1.37, 1.99]**

Heterogeneity: $Chi^2 = 13.05$, $df = 7$ ($P = 0.07$); $I^2 = 46\%$
Test for overall effect: $Z = 5.22$ ($P < 0.00001$)

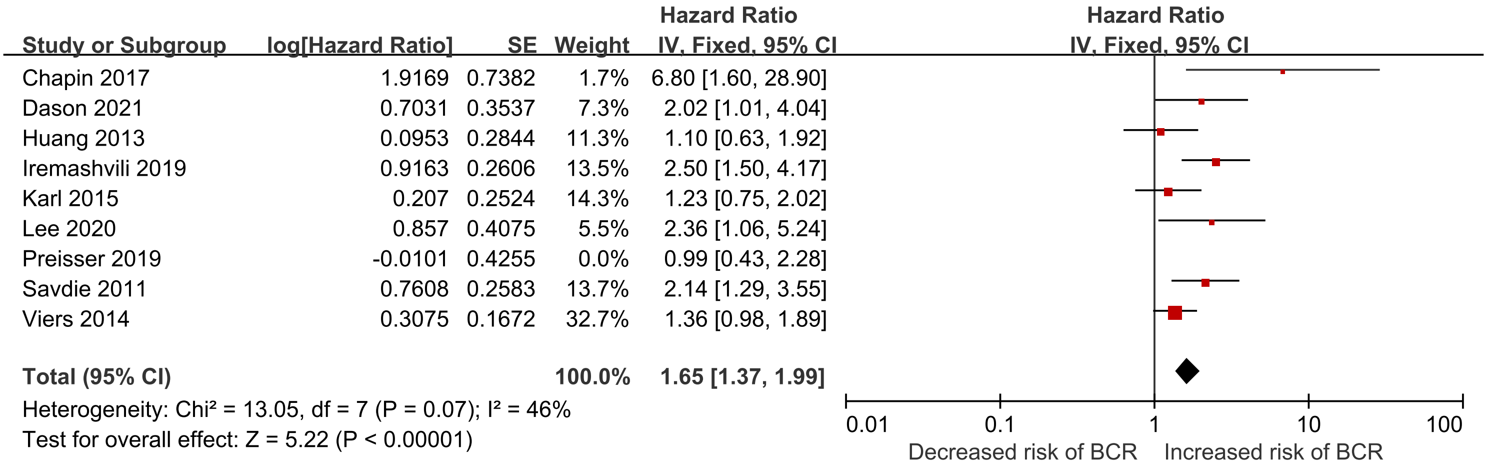

8. Forest plots of studies excluded Savdie's study evaluating the association between PGG and BCR

Study or Subgroup	log[Hazard Ratio]	SE	Weight	Hazard Ratio IV, Fixed, 95% CI
Chapin 2017	1.9169	0.7382	1.8%	6.80 [1.60, 28.90]
Dason 2021	0.7031	0.3537	8.0%	2.02 [1.01, 4.04]
Huang 2013	0.0953	0.2844	12.4%	1.10 [0.63, 1.92]
Iremashvili 2019	0.9163	0.2606	14.7%	2.50 [1.50, 4.17]
Karl 2015	0.207	0.2524	15.7%	1.23 [0.75, 2.02]
Lee 2020	0.857	0.4075	6.0%	2.36 [1.06, 5.24]
Preisser 2019	-0.0101	0.4255	5.5%	0.99 [0.43, 2.28]
Savdie 2011	0.7608	0.2583	0.0%	2.14 [1.29, 3.55]
Viers 2014	0.3075	0.1672	35.8%	1.36 [0.98, 1.89]
**Total (95% CI)**			**100.0%**	**1.54 [1.27, 1.87]**

Heterogeneity: Chi² = 13.01, df = 7 (P = 0.07); I² = 46%
Test for overall effect: Z = 4.32 (P < 0.0001)

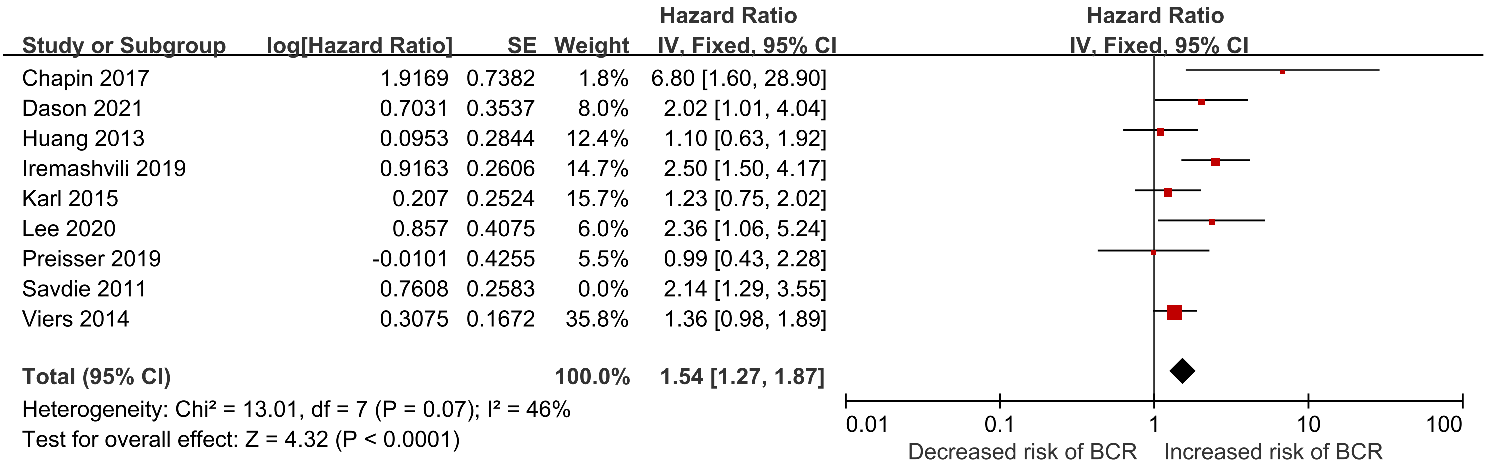

9. Forest plots of studies excluded Viers's study evaluating the association between PGG and BCR

Study or Subgroup	log[Hazard Ratio]	SE	Weight	Hazard Ratio IV, Fixed, 95% CI
Chapin 2017	1.9169	0.7382	2.3%	6.80 [1.60, 28.90]
Dason 2021	0.7031	0.3537	10.1%	2.02 [1.01, 4.04]
Huang 2013	0.0953	0.2844	15.6%	1.10 [0.63, 1.92]
Iremashvili 2019	0.9163	0.2606	18.6%	2.50 [1.50, 4.17]
Karl 2015	0.207	0.2524	19.8%	1.23 [0.75, 2.02]
Lee 2020	0.857	0.4075	7.6%	2.36 [1.06, 5.24]
Preisser 2019	-0.0101	0.4255	7.0%	0.99 [0.43, 2.28]
Savdie 2011	0.7608	0.2583	18.9%	2.14 [1.29, 3.55]
Viers 2014	0.3075	0.1672	0.0%	1.36 [0.98, 1.89]
**Total (95% CI)**			**100.0%**	**1.73 [1.39, 2.16]**

Heterogeneity: Chi² = 12.96, df = 7 (P = 0.07); I² = 46%
Test for overall effect: Z = 4.90 (P < 0.00001)

Hazard Ratio
IV, Fixed, 95% CI

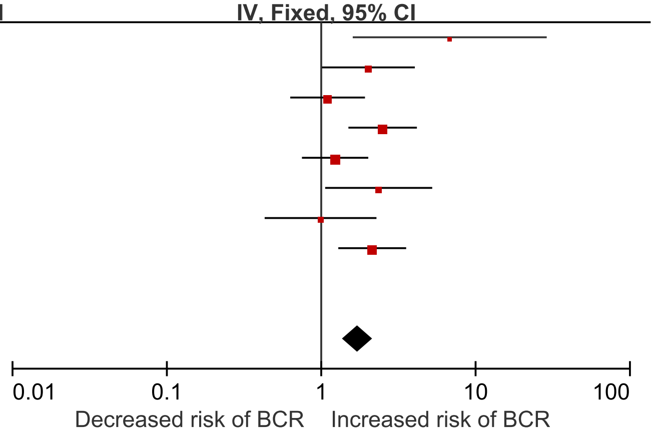

Supplement: S1 File — (PDF) [file pone.0301653.s004.pdf]
