# Supplementary material for: The impact of positive surgical margin parameters and pathological stage on biochemical recurrence after radical prostatectomy: A systematic review and meta-analysis
Source: PLoS One. 2024 Jul 11;19(7):e0301653. doi: 10.1371/journal.pone.0301653 (PMC11239040; doi:10.1371/journal.pone.0301653)

1. Forest plots of studies excluded Dev's study evaluating the association between length of PSM and BCR (<3 VS≥3)

Study or Subgroup	log[Hazard Ratio]	SE	Weight	Hazard Ratio IV, Random, 95% CI
Dev 2015	0.7812	0.2508	0.0%	2.18 [1.34, 3.57]
Kozal 2015	0.2231	0.0988	51.2%	1.25 [1.03, 1.52]
Ploussard 2014	0.6471	0.2245	29.4%	1.91 [1.23, 2.97]
Preisser 2019	0.6575	0.3155	19.4%	1.93 [1.04, 3.58]
**Total (95% CI)**			**100.0%**	**1.54 [1.11, 2.14]**

Heterogeneity: Tau² = 0.05; Chi² = 4.23, df = 2 (P = 0.12); I² = 53%
Test for overall effect: Z = 2.58 (P = 0.010)

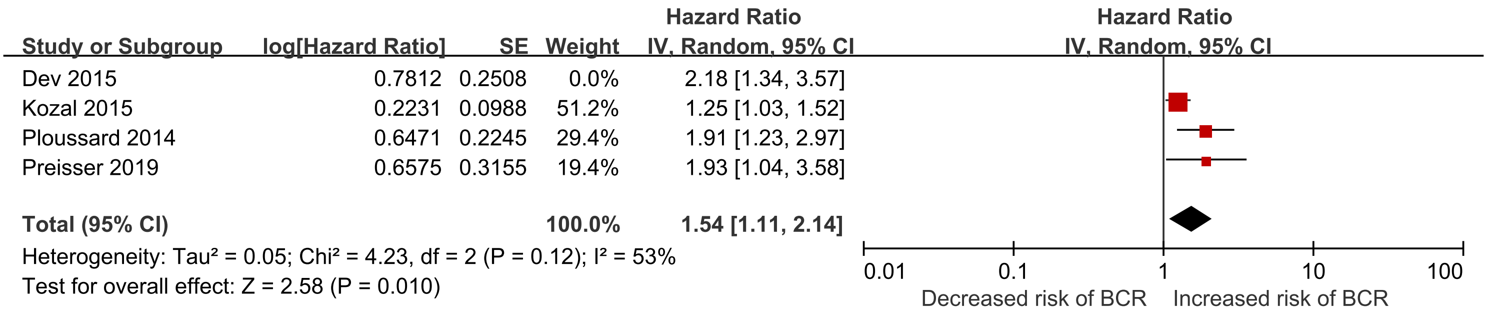

2. Forest plots of studies excluded Kozal's study evaluating the association between length of PSM and BCR (<3 VS≥3)

Study or Subgroup	log[Hazard Ratio]	SE	Weight	Hazard Ratio IV, Fixed, 95% CI
Dev 2015	0.7812	0.2508	34.7%	2.18 [1.34, 3.57]
Kozal 2015	0.2231	0.0988	0.0%	1.25 [1.03, 1.52]
Ploussard 2014	0.6471	0.2245	43.3%	1.91 [1.23, 2.97]
Preisser 2019	0.6575	0.3155	21.9%	1.93 [1.04, 3.58]
**Total (95% CI)**			**100.0%**	**2.01 [1.50, 2.68]**

Heterogeneity: Chi² = 0.18, df = 2 (P = 0.91); I² = 0%

Test for overall effect: Z = 4.71 (P < 0.00001)

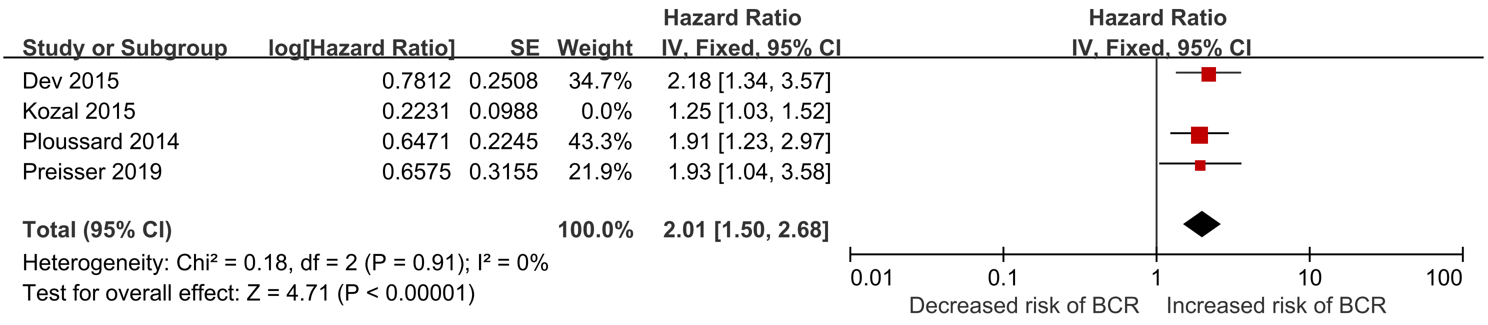

3. Forest plots of studies excluded Ploussard's study evaluating the association between length of PSM and BCR (<3 VS≥3)

Study or Subgroup	log[Hazard Ratio]	SE	Weight	Hazard Ratio IV, Random, 95% CI
Dev 2015	0.7812	0.2508	29.5%	2.18 [1.34, 3.57]
Kozal 2015	0.2231	0.0988	47.1%	1.25 [1.03, 1.52]
Ploussard 2014	0.6471	0.2245	0.0%	1.91 [1.23, 2.97]
Preisser 2019	0.6575	0.3155	23.4%	1.93 [1.04, 3.58]
**Total (95% CI)**			**100.0%**	**1.63 [1.09, 2.44]**

Heterogeneity: $Tau^2 = 0.08$; $Chi^2 = 5.48$, df = 2 (P = 0.06); $I^2 = 64\%$
Test for overall effect: Z = 2.39 (P = 0.02)

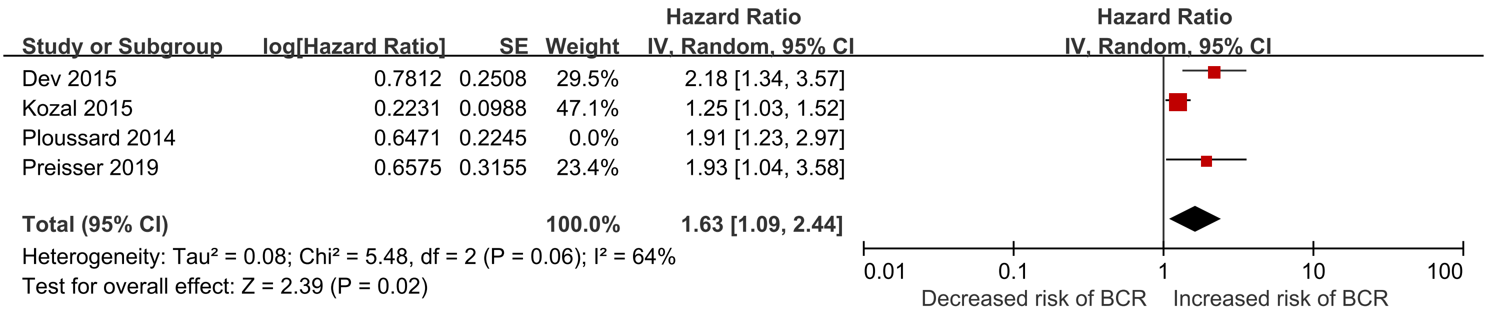

4. Forest plots of studies excluded Preisser's study evaluating the association between length of PSM and BCR (<3 VS≥3)

Study or Subgroup	log[Hazard Ratio]	SE	Weight	Hazard Ratio IV, Random, 95% CI
Dev 2015	0.7812	0.2508	26.9%	2.18 [1.34, 3.57]
Kozal 2015	0.2231	0.0988	43.6%	1.25 [1.03, 1.52]
Ploussard 2014	0.6471	0.2245	29.5%	1.91 [1.23, 2.97]
Preisser 2019	0.6575	0.3155	0.0%	1.93 [1.04, 3.58]
**Total (95% CI)**			**100.0%**	**1.65 [1.13, 2.40]**

Heterogeneity: Tau² = 0.08; Chi² = 6.36, df = 2 (P = 0.04); I² = 69%
Test for overall effect: Z = 2.58 (P = 0.010)

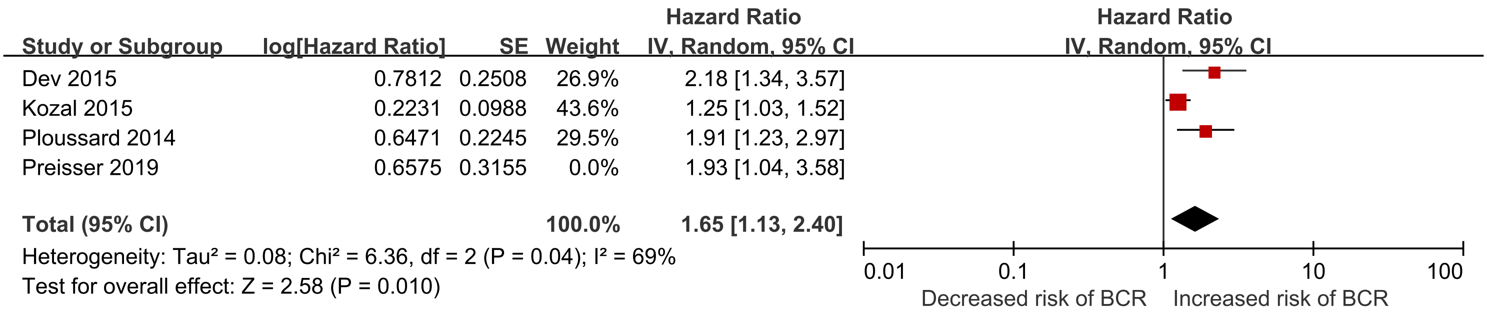

Supplement: S2 File — (PDF) [file pone.0301653.s005.pdf]
