# Supplementary material for: The impact of positive surgical margin parameters and pathological stage on biochemical recurrence after radical prostatectomy: A systematic review and meta-analysis
Source: PLoS One. 2024 Jul 11;19(7):e0301653. doi: 10.1371/journal.pone.0301653 (PMC11239040; doi:10.1371/journal.pone.0301653)

1. Forest plots of studies excluded Dason's study evaluating the association between focality of PSM and BCR

| Study or Subgroup | log[Hazard Ratio] | SE | Weight | Hazard Ratio IV, Fixed, 95% CI |
|---|---|---|---|---|
| Dason 2021 | 0.7031 | 0.2919 | 0.0% | 2.02 [1.14, 3.58] |
| Dev 2015 | -0.0274 | 0.4061 | 2.4% | 0.97 [0.44, 2.16] |
| Ginzburg 2012 | -0.0202 | 0.3642 | 3.0% | 0.98 [0.48, 2.00] |
| Godoy 2009 | 0.4285 | 0.3955 | 2.5% | 1.53 [0.71, 3.33] |
| Lee 2013 | 0.8416 | 0.2469 | 6.5% | 2.32 [1.43, 3.76] |
| May 2011 | -0.0305 | 0.3589 | 3.1% | 0.97 [0.48, 1.96] |
| Porpiglia 2011 | 0.5332 | 0.6257 | 1.0% | 1.70 [0.50, 5.81] |
| Preisser 2019 | 0.0198 | 0.3638 | 3.0% | 1.02 [0.50, 2.08] |
| Resnick 2010 | 0.47 | 0.2247 | 7.8% | 1.60 [1.03, 2.49] |
| Simon 2006 | 0.4055 | 0.2606 | 5.8% | 1.50 [0.90, 2.50] |
| Somford 2011 | 0.0488 | 0.2606 | 5.8% | 1.05 [0.63, 1.75] |
| Song 2017 | 0.27 | 0.1586 | 15.6% | 1.31 [0.96, 1.79] |
| Stephenson 2009 | 0.3365 | 0.123 | 26.0% | 1.40 [1.10, 1.78] |
| Wu 2018 | 0.2311 | 0.1495 | 17.6% | 1.26 [0.94, 1.69] |
| **Total (95% CI)** | | | **100.0%** | **1.35 [1.20, 1.53]** |

Heterogeneity: Chi² = 9.92, df = 12 (P = 0.62); I² = 0%
Test for overall effect: Z = 4.82 (P < 0.00001)

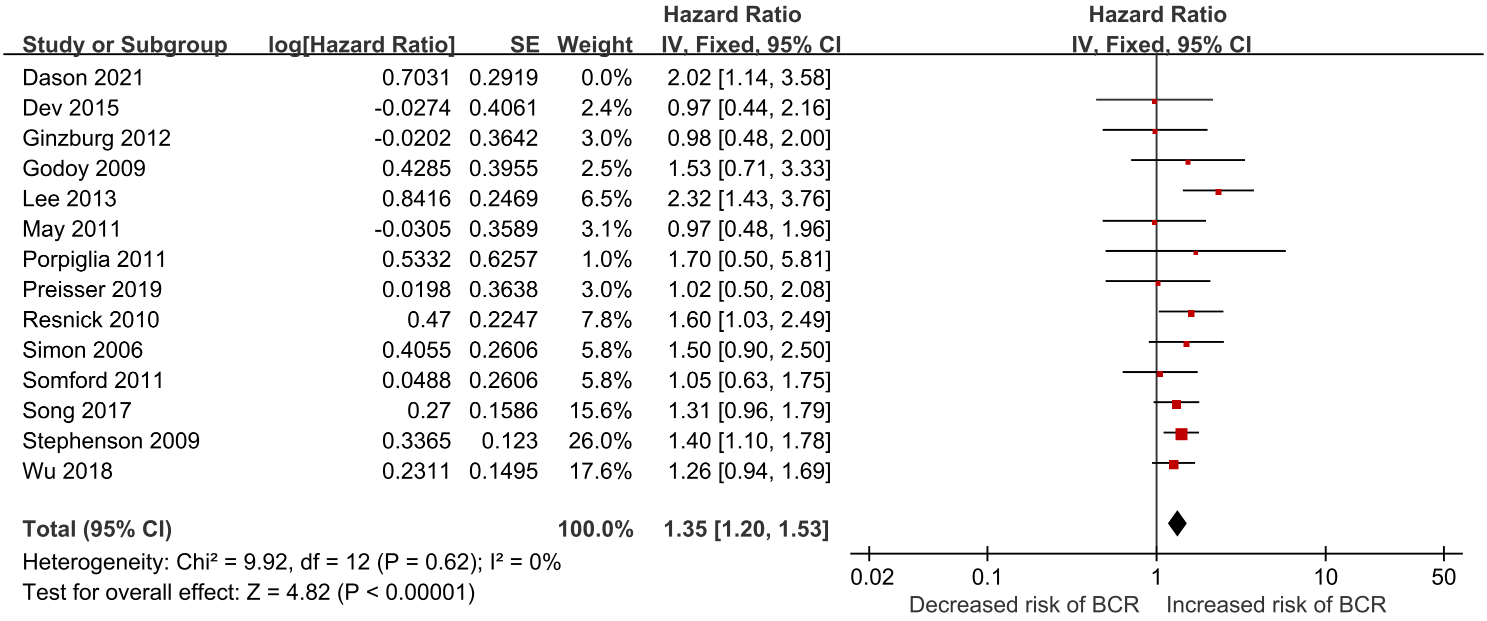

2. Forest plots of  studies excluded Dev's study evaluating the association between focality of PSM and BCR

| Study or Subgroup | log[Hazard Ratio] | SE | Weight | Hazard Ratio IV, Fixed, 95% CI |
|---|---|---|---|---|
| Dason 2021 | 0.7031 | 0.2919 | 4.5% | 2.02 [1.14, 3.58] |
| Dev 2015 | -0.0274 | 0.4061 | 0.0% | 0.97 [0.44, 2.16] |
| Ginzburg 2012 | -0.0202 | 0.3642 | 2.9% | 0.98 [0.48, 2.00] |
| Godoy 2009 | 0.4285 | 0.3955 | 2.5% | 1.53 [0.71, 3.33] |
| Lee 2013 | 0.8416 | 0.2469 | 6.3% | 2.32 [1.43, 3.76] |
| May 2011 | -0.0305 | 0.3589 | 3.0% | 0.97 [0.48, 1.96] |
| Porpiglia 2011 | 0.5332 | 0.6257 | 1.0% | 1.70 [0.50, 5.81] |
| Preisser 2019 | 0.0198 | 0.3638 | 2.9% | 1.02 [0.50, 2.08] |
| Resnick 2010 | 0.47 | 0.2247 | 7.6% | 1.60 [1.03, 2.49] |
| Simon 2006 | 0.4055 | 0.2606 | 5.7% | 1.50 [0.90, 2.50] |
| Somford 2011 | 0.0488 | 0.2606 | 5.7% | 1.05 [0.63, 1.75] |
| Song 2017 | 0.27 | 0.1586 | 15.3% | 1.31 [0.96, 1.79] |
| Stephenson 2009 | 0.3365 | 0.123 | 25.4% | 1.40 [1.10, 1.78] |
| Wu 2018 | 0.2311 | 0.1495 | 17.2% | 1.26 [0.94, 1.69] |
| **Total (95% CI)** | | | **100.0%** | **1.39 [1.23, 1.57]** |

Heterogeneity: Chi² = 10.97, df = 12 (P = 0.53); I² = 0%
Test for overall effect: Z = 5.29 (P < 0.00001)

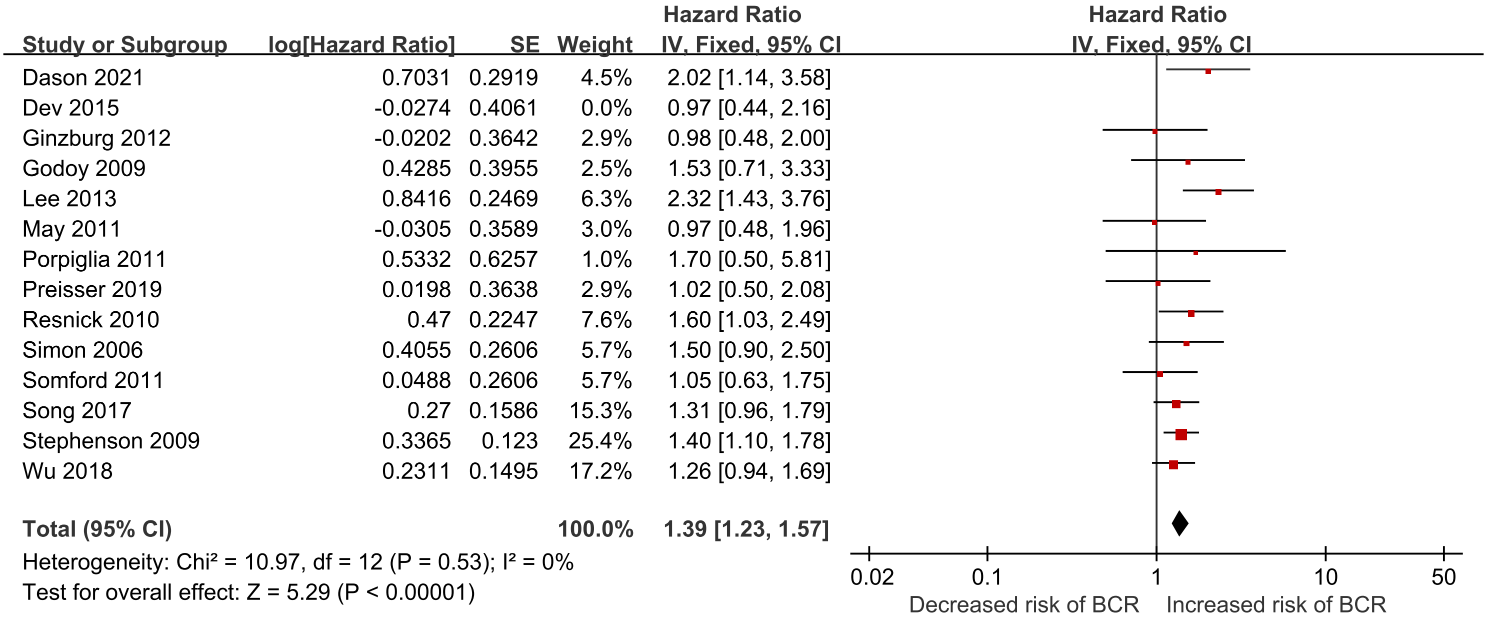

3. Forest plots of  studies excluded Ginzburg's study evaluating the association between focality of PSM and BCR

| Study or Subgroup | log[Hazard Ratio] | SE | Weight | Hazard Ratio IV, Fixed, 95% CI |
|---|---|---|---|---|
| Dason 2021 | 0.7031 | 0.2919 | 4.5% | 2.02 [1.14, 3.58] |
| Dev 2015 | -0.0274 | 0.4061 | 2.3% | 0.97 [0.44, 2.16] |
| Ginzburg 2012 | -0.0202 | 0.3642 | 0.0% | 0.98 [0.48, 2.00] |
| Godoy 2009 | 0.4285 | 0.3955 | 2.5% | 1.53 [0.71, 3.33] |
| Lee 2013 | 0.8416 | 0.2469 | 6.4% | 2.32 [1.43, 3.76] |
| May 2011 | -0.0305 | 0.3589 | 3.0% | 0.97 [0.48, 1.96] |
| Porpiglia 2011 | 0.5332 | 0.6257 | 1.0% | 1.70 [0.50, 5.81] |
| Preisser 2019 | 0.0198 | 0.3638 | 2.9% | 1.02 [0.50, 2.08] |
| Resnick 2010 | 0.47 | 0.2247 | 7.7% | 1.60 [1.03, 2.49] |
| Simon 2006 | 0.4055 | 0.2606 | 5.7% | 1.50 [0.90, 2.50] |
| Somford 2011 | 0.0488 | 0.2606 | 5.7% | 1.05 [0.63, 1.75] |
| Song 2017 | 0.27 | 0.1586 | 15.4% | 1.31 [0.96, 1.79] |
| Stephenson 2009 | 0.3365 | 0.123 | 25.6% | 1.40 [1.10, 1.78] |
| Wu 2018 | 0.2311 | 0.1495 | 17.3% | 1.26 [0.94, 1.69] |
| **Total (95% CI)** | | | **100.0%** | **1.39 [1.23, 1.57]** |

Heterogeneity: Chi² = 10.83, df = 12 (P = 0.54); I² = 0%
Test for overall effect: Z = 5.30 (P < 0.00001)

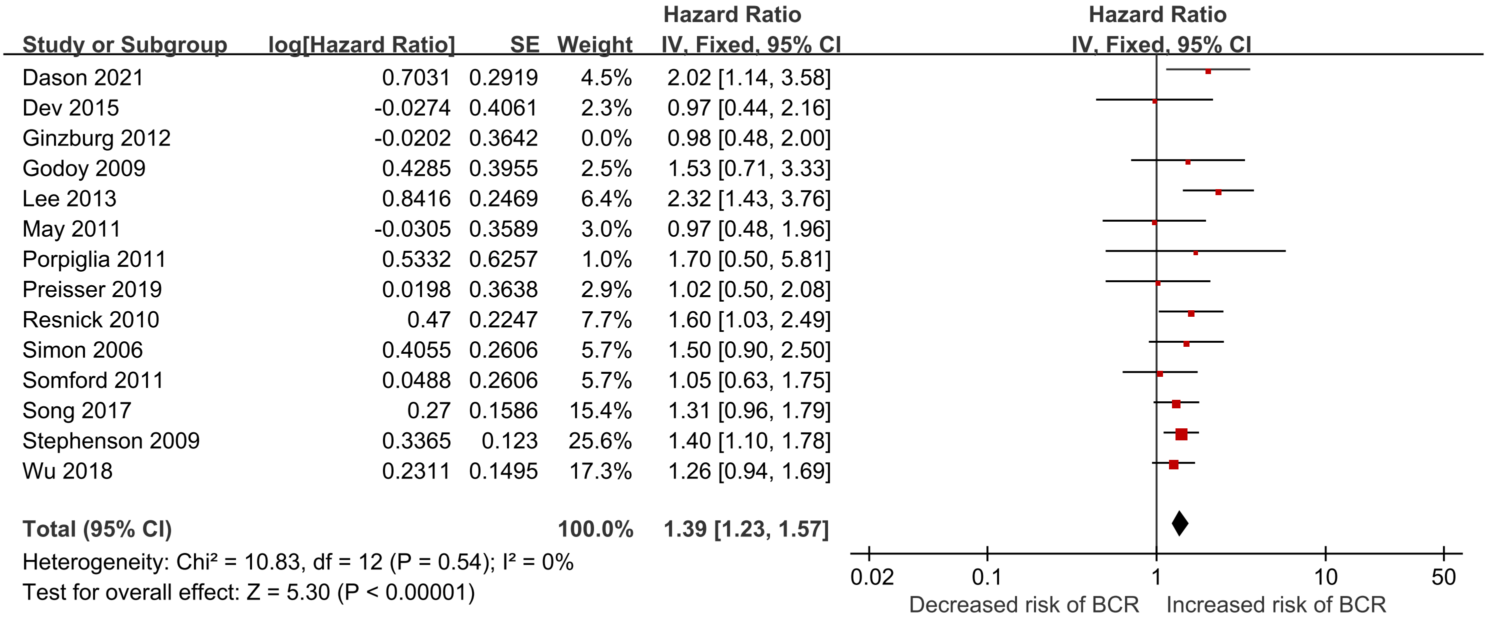

4. Forest plots of  studies excluded Godoy's study evaluating the association between focality of PSM and BCR

| Study or Subgroup | log[Hazard Ratio] | SE | Weight | Hazard Ratio IV, Fixed, 95% CI |
|---|---|---|---|---|
| Dason 2021 | 0.7031 | 0.2919 | 4.5% | 2.02 [1.14, 3.58] |
| Dev 2015 | -0.0274 | 0.4061 | 2.3% | 0.97 [0.44, 2.16] |
| Ginzburg 2012 | -0.0202 | 0.3642 | 2.9% | 0.98 [0.48, 2.00] |
| Godoy 2009 | 0.4285 | 0.3955 | 0.0% | 1.53 [0.71, 3.33] |
| Lee 2013 | 0.8416 | 0.2469 | 6.3% | 2.32 [1.43, 3.76] |
| May 2011 | -0.0305 | 0.3589 | 3.0% | 0.97 [0.48, 1.96] |
| Porpiglia 2011 | 0.5332 | 0.6257 | 1.0% | 1.70 [0.50, 5.81] |
| Preisser 2019 | 0.0198 | 0.3638 | 2.9% | 1.02 [0.50, 2.08] |
| Resnick 2010 | 0.47 | 0.2247 | 7.6% | 1.60 [1.03, 2.49] |
| Simon 2006 | 0.4055 | 0.2606 | 5.7% | 1.50 [0.90, 2.50] |
| Somford 2011 | 0.0488 | 0.2606 | 5.7% | 1.05 [0.63, 1.75] |
| Song 2017 | 0.27 | 0.1586 | 15.3% | 1.31 [0.96, 1.79] |
| Stephenson 2009 | 0.3365 | 0.123 | 25.5% | 1.40 [1.10, 1.78] |
| Wu 2018 | 0.2311 | 0.1495 | 17.2% | 1.26 [0.94, 1.69] |
| **Total (95% CI)** | | | **100.0%** | **1.37 [1.22, 1.55]** |

Heterogeneity: Chi² = 11.65, df = 12 (P = 0.47); I² = 0%

Test for overall effect: Z = 5.11 (P < 0.00001)

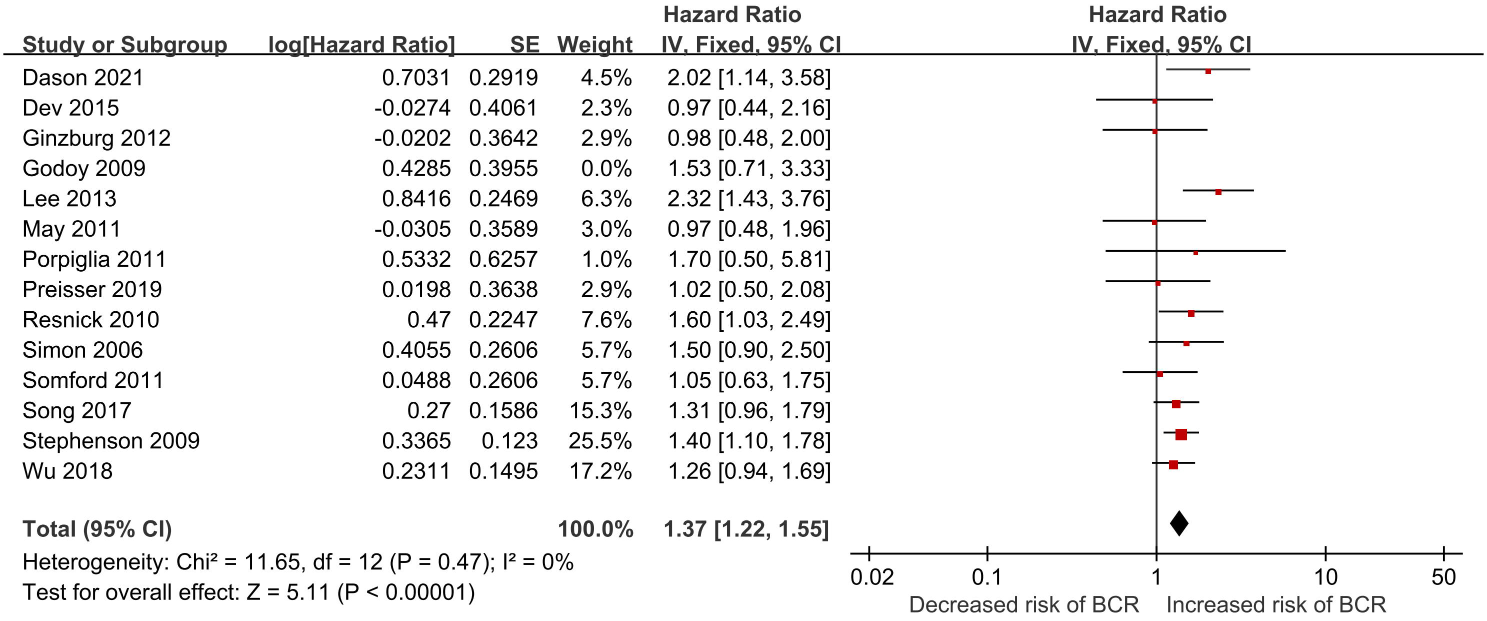

5. Forest plots of  studies excluded Lee's study evaluating the association between focality of PSM and BCR

| Study or Subgroup | log[Hazard Ratio] | SE | Weight | Hazard Ratio IV, Fixed, 95% CI |
|---|---|---|---|---|
| Dason 2021 | 0.7031 | 0.2919 | 4.7% | 2.02 [1.14, 3.58] |
| Dev 2015 | -0.0274 | 0.4061 | 2.4% | 0.97 [0.44, 2.16] |
| Ginzburg 2012 | -0.0202 | 0.3642 | 3.0% | 0.98 [0.48, 2.00] |
| Godoy 2009 | 0.4285 | 0.3955 | 2.6% | 1.53 [0.71, 3.33] |
| Lee 2013 | 0.8416 | 0.2469 | 0.0% | 2.32 [1.43, 3.76] |
| May 2011 | -0.0305 | 0.3589 | 3.1% | 0.97 [0.48, 1.96] |
| Porpiglia 2011 | 0.5332 | 0.6257 | 1.0% | 1.70 [0.50, 5.81] |
| Preisser 2019 | 0.0198 | 0.3638 | 3.0% | 1.02 [0.50, 2.08] |
| Resnick 2010 | 0.47 | 0.2247 | 7.9% | 1.60 [1.03, 2.49] |
| Simon 2006 | 0.4055 | 0.2606 | 5.9% | 1.50 [0.90, 2.50] |
| Somford 2011 | 0.0488 | 0.2606 | 5.9% | 1.05 [0.63, 1.75] |
| Song 2017 | 0.27 | 0.1586 | 15.9% | 1.31 [0.96, 1.79] |
| Stephenson 2009 | 0.3365 | 0.123 | 26.5% | 1.40 [1.10, 1.78] |
| Wu 2018 | 0.2311 | 0.1495 | 17.9% | 1.26 [0.94, 1.69] |
| **Total (95% CI)** | | | **100.0%** | **1.33 [1.18, 1.51]** |

Heterogeneity: Chi² = 6.97, df = 12 (P = 0.86); I² = 0%

Test for overall effect: Z = 4.51 (P < 0.00001)

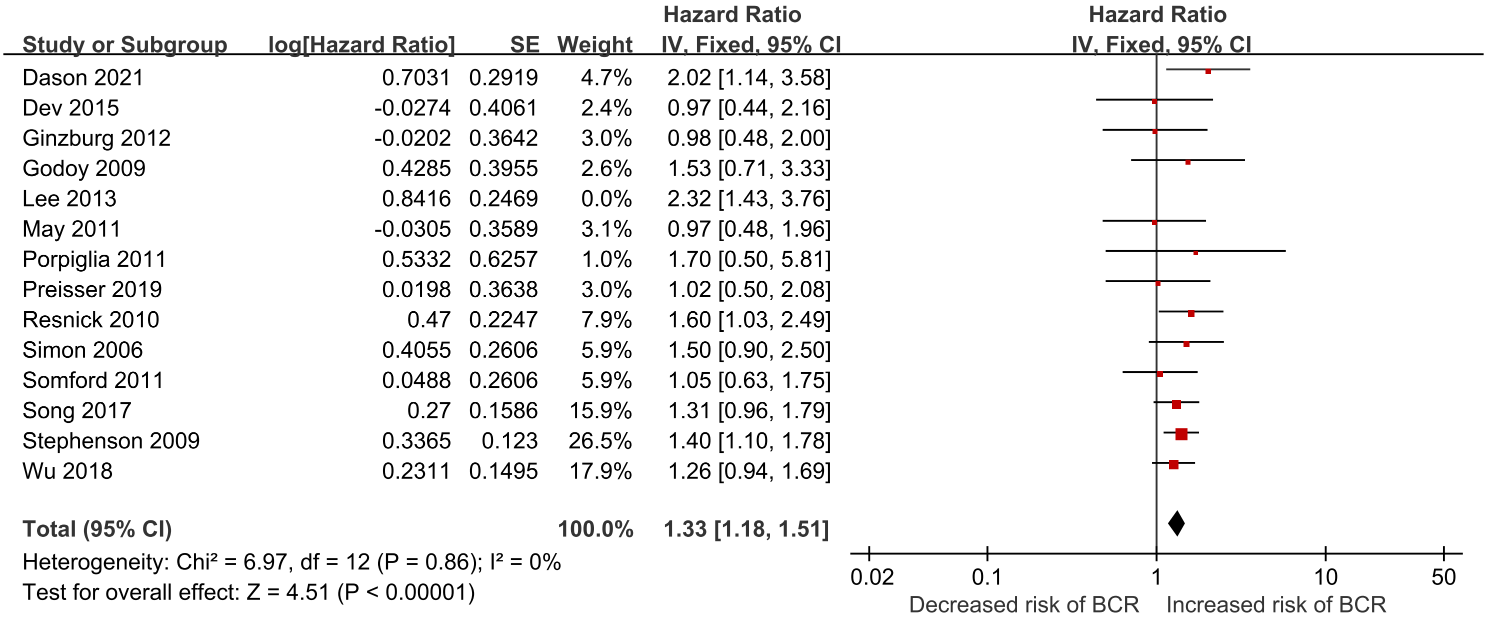

6. Forest plots of  studies excluded May's study evaluating the association between focality of PSM and BCR

| Study or Subgroup | log[Hazard Ratio] | SE | Weight | Hazard Ratio IV, Fixed, 95% CI |
|---|---|---|---|---|
| Dason 2021 | 0.7031 | 0.2919 | 4.5% | 2.02 [1.14, 3.58] |
| Dev 2015 | -0.0274 | 0.4061 | 2.3% | 0.97 [0.44, 2.16] |
| Ginzburg 2012 | -0.0202 | 0.3642 | 2.9% | 0.98 [0.48, 2.00] |
| Godoy 2009 | 0.4285 | 0.3955 | 2.5% | 1.53 [0.71, 3.33] |
| Lee 2013 | 0.8416 | 0.2469 | 6.4% | 2.32 [1.43, 3.76] |
| May 2011 | -0.0305 | 0.3589 | 0.0% | 0.97 [0.48, 1.96] |
| Porpiglia 2011 | 0.5332 | 0.6257 | 1.0% | 1.70 [0.50, 5.81] |
| Preisser 2019 | 0.0198 | 0.3638 | 2.9% | 1.02 [0.50, 2.08] |
| Resnick 2010 | 0.47 | 0.2247 | 7.7% | 1.60 [1.03, 2.49] |
| Simon 2006 | 0.4055 | 0.2606 | 5.7% | 1.50 [0.90, 2.50] |
| Somford 2011 | 0.0488 | 0.2606 | 5.7% | 1.05 [0.63, 1.75] |
| Song 2017 | 0.27 | 0.1586 | 15.4% | 1.31 [0.96, 1.79] |
| Stephenson 2009 | 0.3365 | 0.123 | 25.6% | 1.40 [1.10, 1.78] |
| Wu 2018 | 0.2311 | 0.1495 | 17.3% | 1.26 [0.94, 1.69] |
| **Total (95% CI)** | | | **100.0%** | **1.39 [1.23, 1.57]** |

Heterogeneity: Chi² = 10.74, df = 12 (P = 0.55); I² = 0%
Test for overall effect: Z = 5.31 (P < 0.00001)

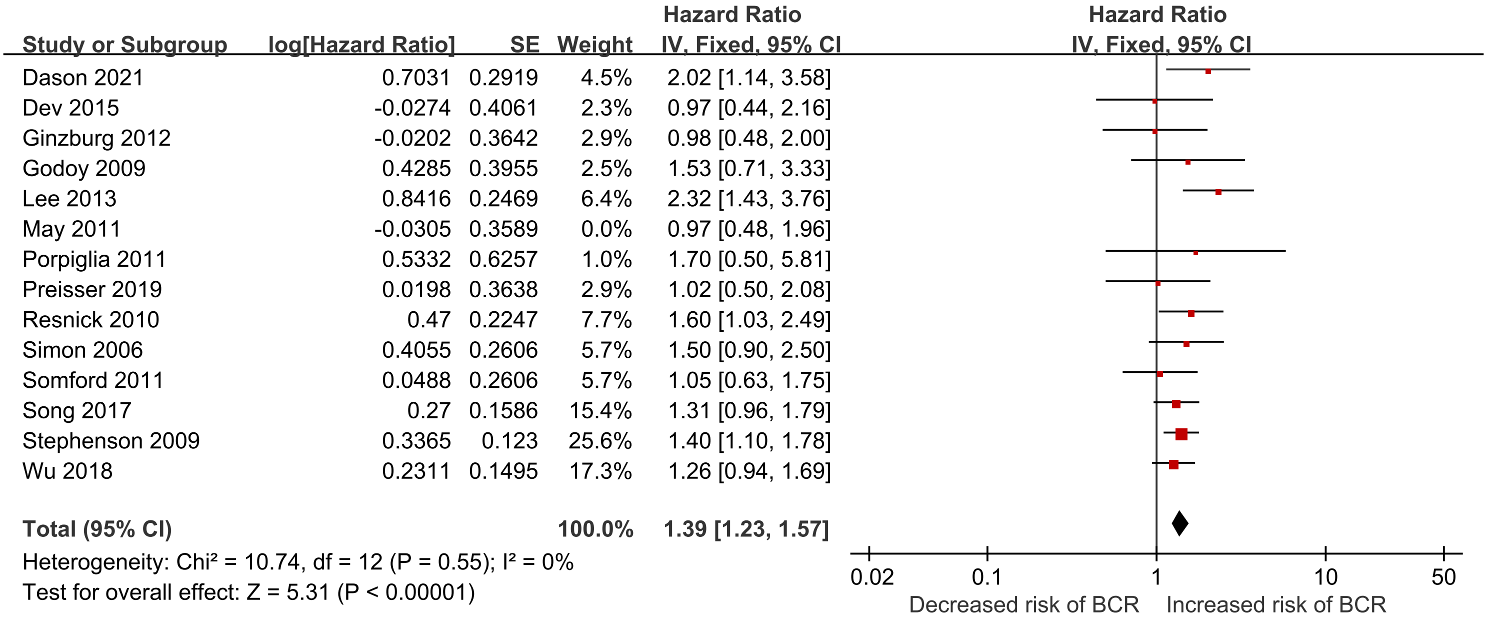

7. Forest plots of studies excluded Porpiglia's study evaluating the association between focality of PSM and BCR

| Study or Subgroup | log[Hazard Ratio] | SE | Weight | Hazard Ratio IV, Fixed, 95% CI |
|---|---|---|---|---|
| Dason 2021 | 0.7031 | 0.2919 | 4.5% | 2.02 [1.14, 3.58] |
| Dev 2015 | -0.0274 | 0.4061 | 2.3% | 0.97 [0.44, 2.16] |
| Ginzburg 2012 | -0.0202 | 0.3642 | 2.9% | 0.98 [0.48, 2.00] |
| Godoy 2009 | 0.4285 | 0.3955 | 2.4% | 1.53 [0.71, 3.33] |
| Lee 2013 | 0.8416 | 0.2469 | 6.2% | 2.32 [1.43, 3.76] |
| May 2011 | -0.0305 | 0.3589 | 2.9% | 0.97 [0.48, 1.96] |
| Porpiglia 2011 | 0.5332 | 0.6257 | 0.0% | 1.70 [0.50, 5.81] |
| Preisser 2019 | 0.0198 | 0.3638 | 2.9% | 1.02 [0.50, 2.08] |
| Resnick 2010 | 0.47 | 0.2247 | 7.5% | 1.60 [1.03, 2.49] |
| Simon 2006 | 0.4055 | 0.2606 | 5.6% | 1.50 [0.90, 2.50] |
| Somford 2011 | 0.0488 | 0.2606 | 5.6% | 1.05 [0.63, 1.75] |
| Song 2017 | 0.27 | 0.1586 | 15.1% | 1.31 [0.96, 1.79] |
| Stephenson 2009 | 0.3365 | 0.123 | 25.1% | 1.40 [1.10, 1.78] |
| Wu 2018 | 0.2311 | 0.1495 | 17.0% | 1.26 [0.94, 1.69] |
| **Total (95% CI)** | | | **100.0%** | **1.37 [1.22, 1.55]** |

Heterogeneity: Chi² = 11.61, df = 12 (P = 0.48); I² = 0%

Test for overall effect: Z = 5.16 (P < 0.00001)

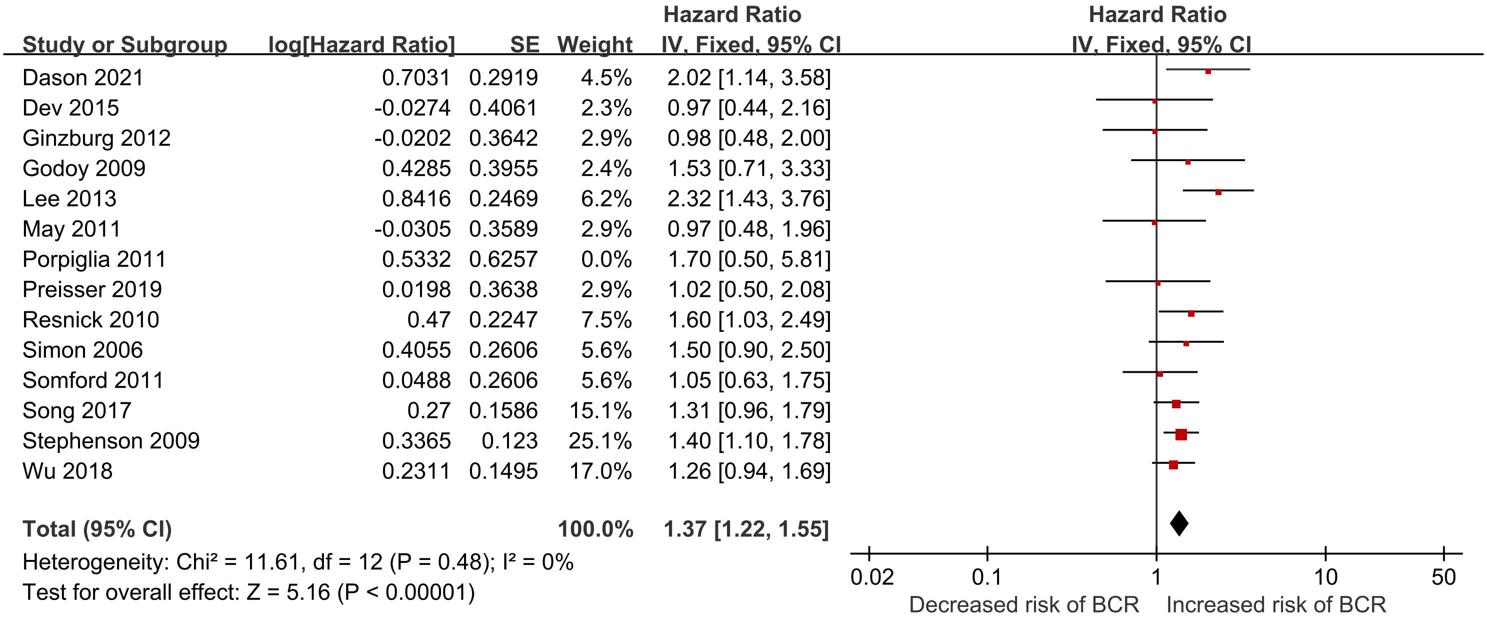

8. Forest plots of studies excluded Preisser's study evaluating the association between focality of PSM and BCR

| Study or Subgroup | log[Hazard Ratio] | SE | Weight | Hazard Ratio IV, Fixed, 95% CI |
|---|---|---|---|---|
| Dason 2021 | 0.7031 | 0.2919 | 4.5% | 2.02 [1.14, 3.58] |
| Dev 2015 | -0.0274 | 0.4061 | 2.3% | 0.97 [0.44, 2.16] |
| Ginzburg 2012 | -0.0202 | 0.3642 | 2.9% | 0.98 [0.48, 2.00] |
| Godoy 2009 | 0.4285 | 0.3955 | 2.5% | 1.53 [0.71, 3.33] |
| Lee 2013 | 0.8416 | 0.2469 | 6.4% | 2.32 [1.43, 3.76] |
| May 2011 | -0.0305 | 0.3589 | 3.0% | 0.97 [0.48, 1.96] |
| Porpiglia 2011 | 0.5332 | 0.6257 | 1.0% | 1.70 [0.50, 5.81] |
| Preisser 2019 | 0.0198 | 0.3638 | 0.0% | 1.02 [0.50, 2.08] |
| Resnick 2010 | 0.47 | 0.2247 | 7.7% | 1.60 [1.03, 2.49] |
| Simon 2006 | 0.4055 | 0.2606 | 5.7% | 1.50 [0.90, 2.50] |
| Somford 2011 | 0.0488 | 0.2606 | 5.7% | 1.05 [0.63, 1.75] |
| Song 2017 | 0.27 | 0.1586 | 15.4% | 1.31 [0.96, 1.79] |
| Stephenson 2009 | 0.3365 | 0.123 | 25.6% | 1.40 [1.10, 1.78] |
| Wu 2018 | 0.2311 | 0.1495 | 17.3% | 1.26 [0.94, 1.69] |
| **Total (95% CI)** | | | **100.0%** | **1.39 [1.23, 1.57]** |

Heterogeneity: Chi² = 11.02, df = 12 (P = 0.53); I² = 0%

Test for overall effect: Z = 5.28 (P < 0.00001)

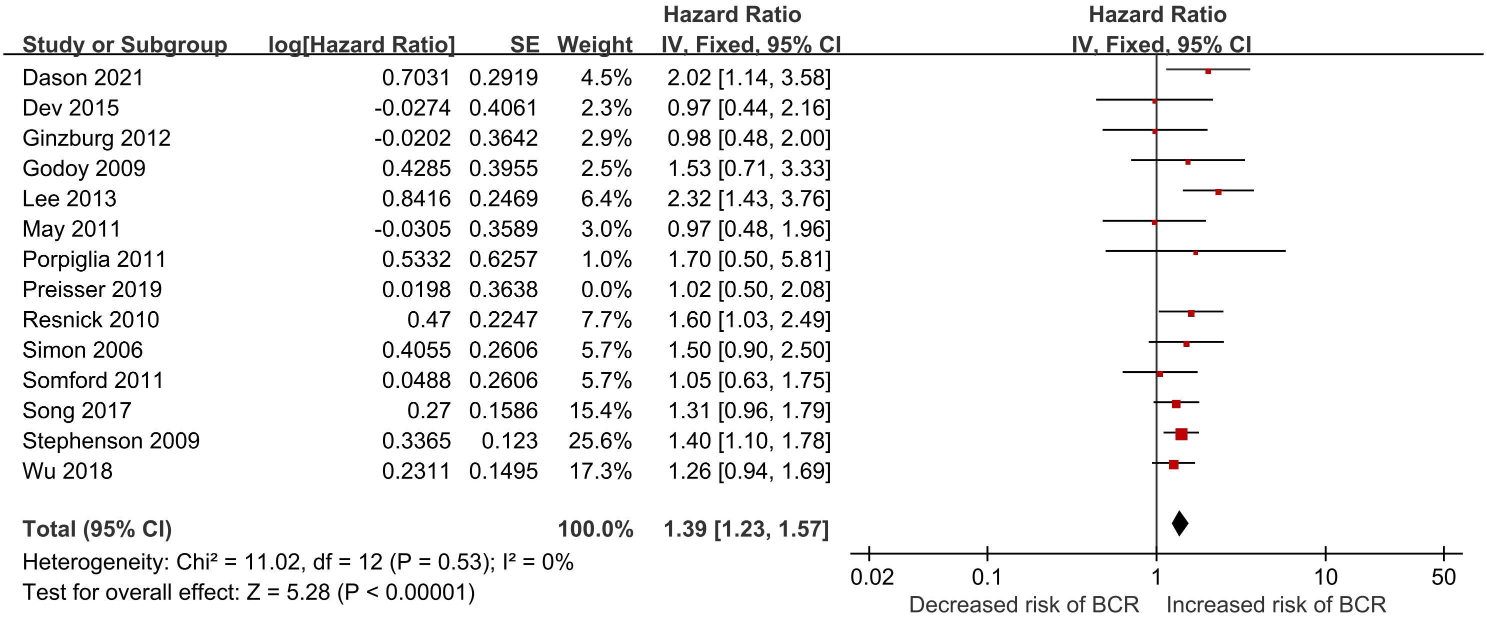

9. Forest plots of studies excluded Resnick's study evaluating the association between focality of PSM and BCR

| Study or Subgroup | log[Hazard Ratio] | SE | Weight | Hazard Ratio IV, Fixed, 95% CI |
|---|---|---|---|---|
| Dason 2021 | 0.7031 | 0.2919 | 4.8% | 2.02 [1.14, 3.58] |
| Dev 2015 | -0.0274 | 0.4061 | 2.5% | 0.97 [0.44, 2.16] |
| Ginzburg 2012 | -0.0202 | 0.3642 | 3.1% | 0.98 [0.48, 2.00] |
| Godoy 2009 | 0.4285 | 0.3955 | 2.6% | 1.53 [0.71, 3.33] |
| Lee 2013 | 0.8416 | 0.2469 | 6.7% | 2.32 [1.43, 3.76] |
| May 2011 | -0.0305 | 0.3589 | 3.2% | 0.97 [0.48, 1.96] |
| Porpiglia 2011 | 0.5332 | 0.6257 | 1.0% | 1.70 [0.50, 5.81] |
| Preisser 2019 | 0.0198 | 0.3638 | 3.1% | 1.02 [0.50, 2.08] |
| Resnick 2010 | 0.47 | 0.2247 | 0.0% | 1.60 [1.03, 2.49] |
| Simon 2006 | 0.4055 | 0.2606 | 6.0% | 1.50 [0.90, 2.50] |
| Somford 2011 | 0.0488 | 0.2606 | 6.0% | 1.05 [0.63, 1.75] |
| Song 2017 | 0.27 | 0.1586 | 16.2% | 1.31 [0.96, 1.79] |
| Stephenson 2009 | 0.3365 | 0.123 | 26.9% | 1.40 [1.10, 1.78] |
| Wu 2018 | 0.2311 | 0.1495 | 18.2% | 1.26 [0.94, 1.69] |
| **Total (95% CI)** | | | **100.0%** | **1.36 [1.20, 1.54]** |

Heterogeneity: Chi² = 11.24, df = 12 (P = 0.51); I² = 0%
Test for overall effect: Z = 4.83 (P < 0.00001)

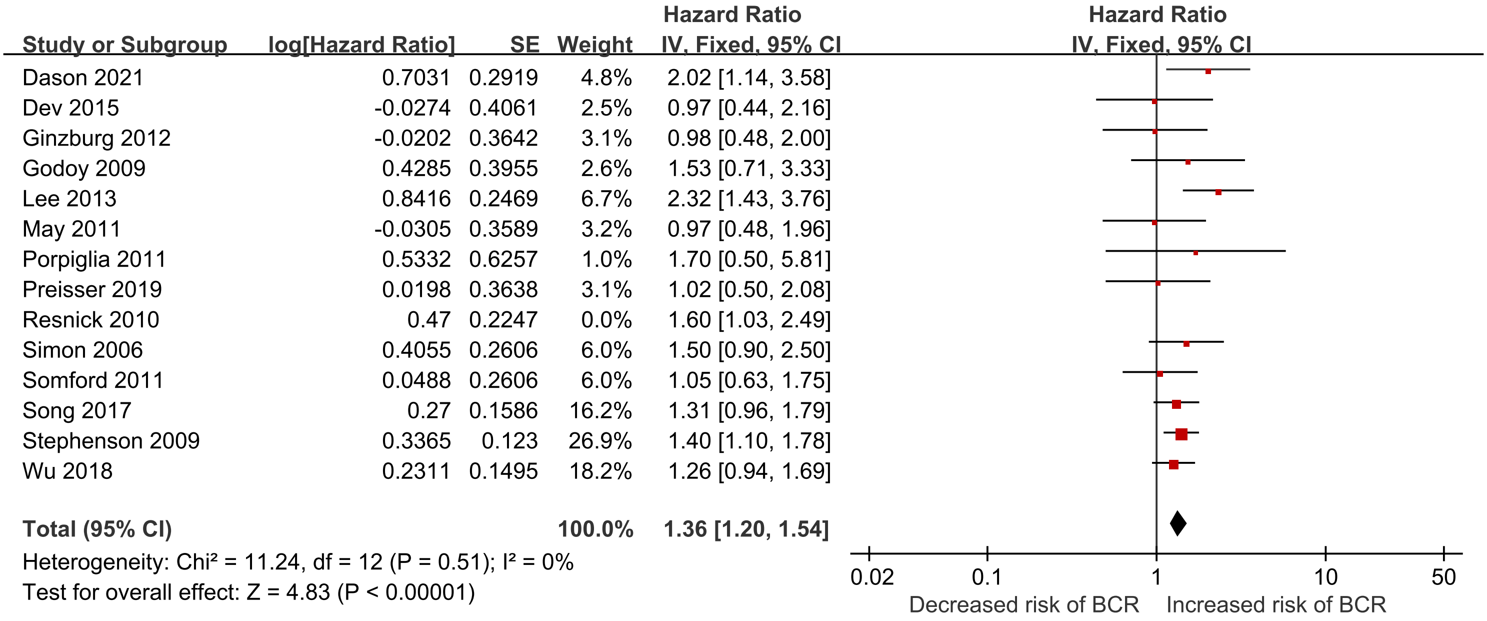

10. Forest plots of  studies excluded Simon's study evaluating the association between focality of PSM and BCR

| Study or Subgroup | log[Hazard Ratio] | SE | Weight | Hazard Ratio<br>IV, Fixed, 95% CI |
|---|---|---|---|---|
| Dason 2021 | 0.7031 | 0.2919 | 4.7% | 2.02 [1.14, 3.58] |
| Dev 2015 | -0.0274 | 0.4061 | 2.4% | 0.97 [0.44, 2.16] |
| Ginzburg 2012 | -0.0202 | 0.3642 | 3.0% | 0.98 [0.48, 2.00] |
| Godoy 2009 | 0.4285 | 0.3955 | 2.5% | 1.53 [0.71, 3.33] |
| Lee 2013 | 0.8416 | 0.2469 | 6.5% | 2.32 [1.43, 3.76] |
| May 2011 | -0.0305 | 0.3589 | 3.1% | 0.97 [0.48, 1.96] |
| Porpiglia 2011 | 0.5332 | 0.6257 | 1.0% | 1.70 [0.50, 5.81] |
| Preisser 2019 | 0.0198 | 0.3638 | 3.0% | 1.02 [0.50, 2.08] |
| Resnick 2010 | 0.47 | 0.2247 | 7.9% | 1.60 [1.03, 2.49] |
| Simon 2006 | 0.4055 | 0.2606 | 0.0% | 1.50 [0.90, 2.50] |
| Somford 2011 | 0.0488 | 0.2606 | 5.9% | 1.05 [0.63, 1.75] |
| Song 2017 | 0.27 | 0.1586 | 15.8% | 1.31 [0.96, 1.79] |
| Stephenson 2009 | 0.3365 | 0.123 | 26.3% | 1.40 [1.10, 1.78] |
| Wu 2018 | 0.2311 | 0.1495 | 17.8% | 1.26 [0.94, 1.69] |
| **Total (95% CI)** | | | **100.0%** | **1.37 [1.21, 1.55]** |

Heterogeneity: Chi² = 11.61, df = 12 (P = 0.48); $I^2$ = 0%

Test for overall effect: Z = 4.99 (P < 0.00001)

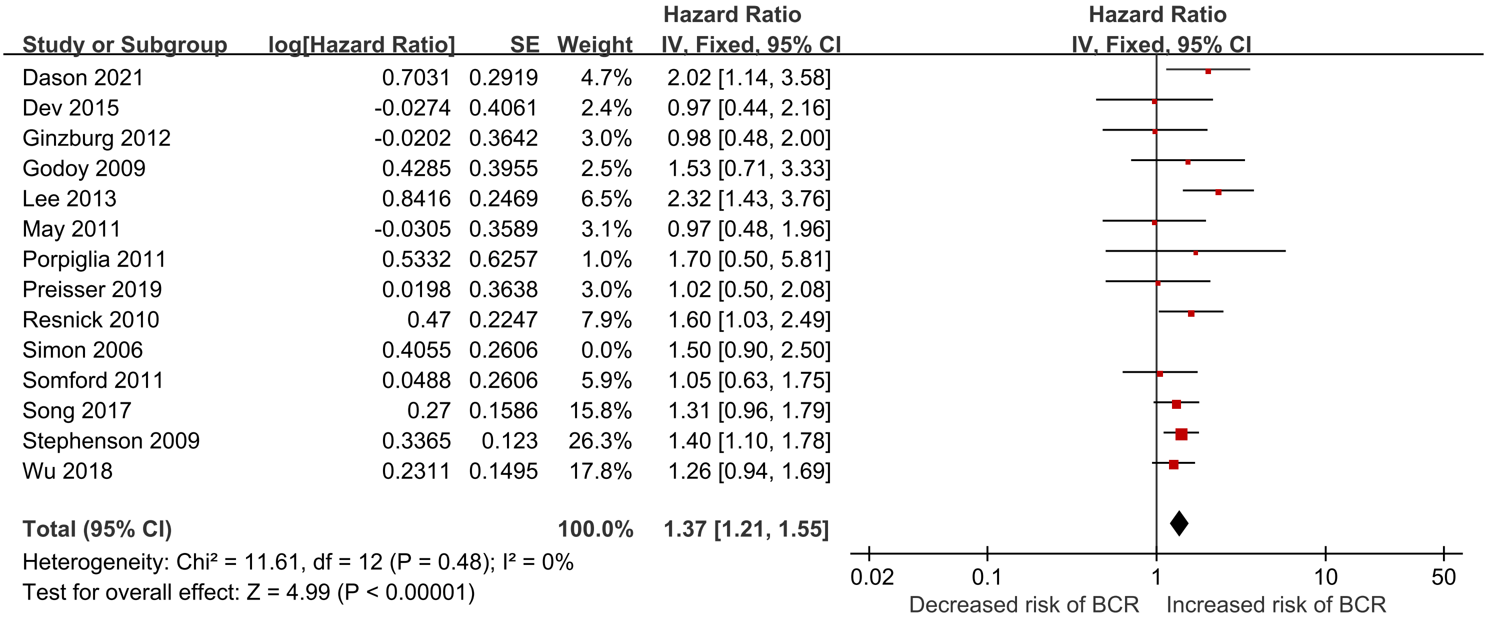

11. Forest plots of  studies excluded Somford's study evaluating the association between focality of PSM and BCR

| Study or Subgroup | log[Hazard Ratio] | SE | Weight | Hazard Ratio IV, Fixed, 95% CI |
|---|---|---|---|---|
| Dason 2021 | 0.7031 | 0.2919 | 4.7% | 2.02 [1.14, 3.58] |
| Dev 2015 | -0.0274 | 0.4061 | 2.4% | 0.97 [0.44, 2.16] |
| Ginzburg 2012 | -0.0202 | 0.3642 | 3.0% | 0.98 [0.48, 2.00] |
| Godoy 2009 | 0.4285 | 0.3955 | 2.5% | 1.53 [0.71, 3.33] |
| Lee 2013 | 0.8416 | 0.2469 | 6.5% | 2.32 [1.43, 3.76] |
| May 2011 | -0.0305 | 0.3589 | 3.1% | 0.97 [0.48, 1.96] |
| Porpiglia 2011 | 0.5332 | 0.6257 | 1.0% | 1.70 [0.50, 5.81] |
| Preisser 2019 | 0.0198 | 0.3638 | 3.0% | 1.02 [0.50, 2.08] |
| Resnick 2010 | 0.47 | 0.2247 | 7.9% | 1.60 [1.03, 2.49] |
| Simon 2006 | 0.4055 | 0.2606 | 5.9% | 1.50 [0.90, 2.50] |
| Somford 2011 | 0.0488 | 0.2606 | 0.0% | 1.05 [0.63, 1.75] |
| Song 2017 | 0.27 | 0.1586 | 15.8% | 1.31 [0.96, 1.79] |
| Stephenson 2009 | 0.3365 | 0.123 | 26.3% | 1.40 [1.10, 1.78] |
| Wu 2018 | 0.2311 | 0.1495 | 17.8% | 1.26 [0.94, 1.69] |
| **Total (95% CI)** | | | **100.0%** | **1.40 [1.24, 1.58]** |

Heterogeneity: Chi² = 10.58, df = 12 (P = 0.57); I² = 0%

Test for overall effect: Z = 5.32 (P < 0.00001)

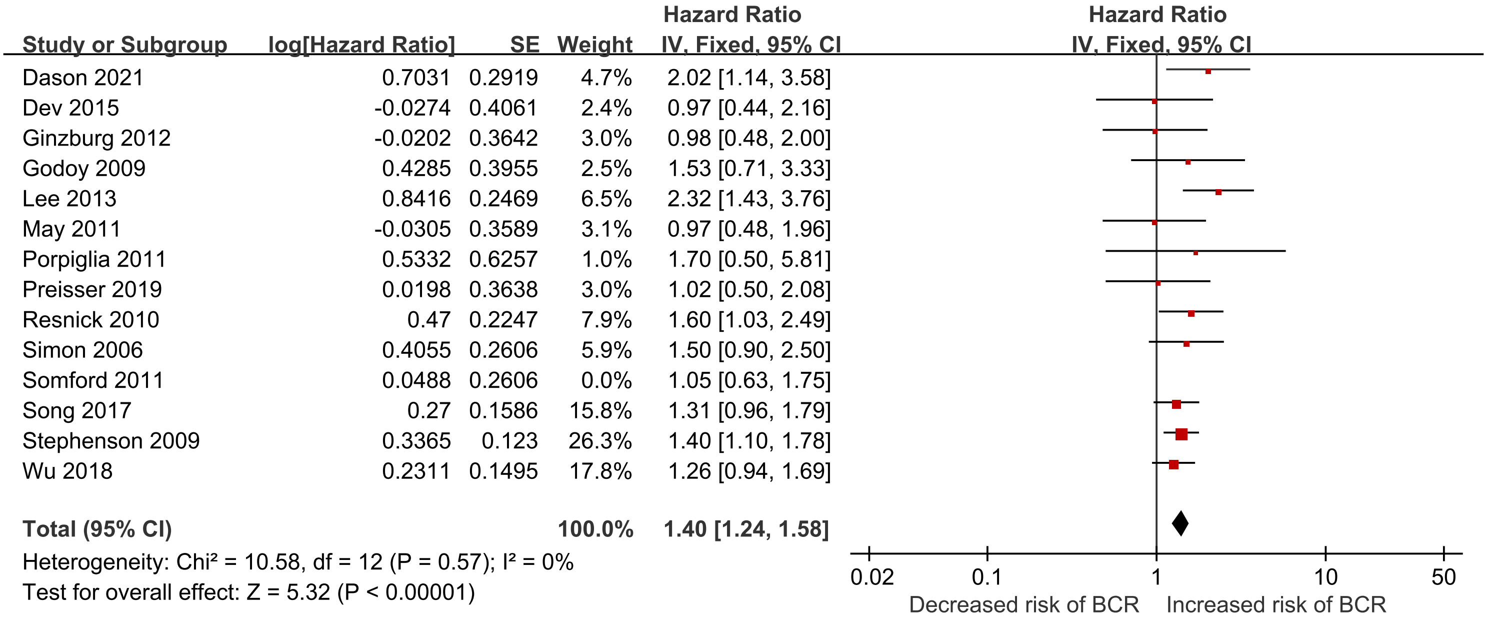

12. Forest plots of  studies excluded Song's study evaluating the association between focality of PSM and BCR

| Study or Subgroup | log[Hazard Ratio] | SE | Weight | Hazard Ratio IV, Fixed, 95% CI |
|---|---|---|---|---|
| Dason 2021 | 0.7031 | 0.2919 | 5.2% | 2.02 [1.14, 3.58] |
| Dev 2015 | -0.0274 | 0.4061 | 2.7% | 0.97 [0.44, 2.16] |
| Ginzburg 2012 | -0.0202 | 0.3642 | 3.3% | 0.98 [0.48, 2.00] |
| Godoy 2009 | 0.4285 | 0.3955 | 2.8% | 1.53 [0.71, 3.33] |
| Lee 2013 | 0.8416 | 0.2469 | 7.3% | 2.32 [1.43, 3.76] |
| May 2011 | -0.0305 | 0.3589 | 3.4% | 0.97 [0.48, 1.96] |
| Porpiglia 2011 | 0.5332 | 0.6257 | 1.1% | 1.70 [0.50, 5.81] |
| Preisser 2019 | 0.0198 | 0.3638 | 3.3% | 1.02 [0.50, 2.08] |
| Resnick 2010 | 0.47 | 0.2247 | 8.8% | 1.60 [1.03, 2.49] |
| Simon 2006 | 0.4055 | 0.2606 | 6.5% | 1.50 [0.90, 2.50] |
| Somford 2011 | 0.0488 | 0.2606 | 6.5% | 1.05 [0.63, 1.75] |
| Song 2017 | 0.27 | 0.1586 | 0.0% | 1.31 [0.96, 1.79] |
| Stephenson 2009 | 0.3365 | 0.123 | 29.2% | 1.40 [1.10, 1.78] |
| Wu 2018 | 0.2311 | 0.1495 | 19.8% | 1.26 [0.94, 1.69] |
| **Total (95% CI)** | | | **100.0%** | **1.39 [1.22, 1.58]** |

Heterogeneity: Chi² = 11.61, df = 12 (P = 0.48); $I^2$ = 0%
Test for overall effect: Z = 4.94 (P < 0.00001)

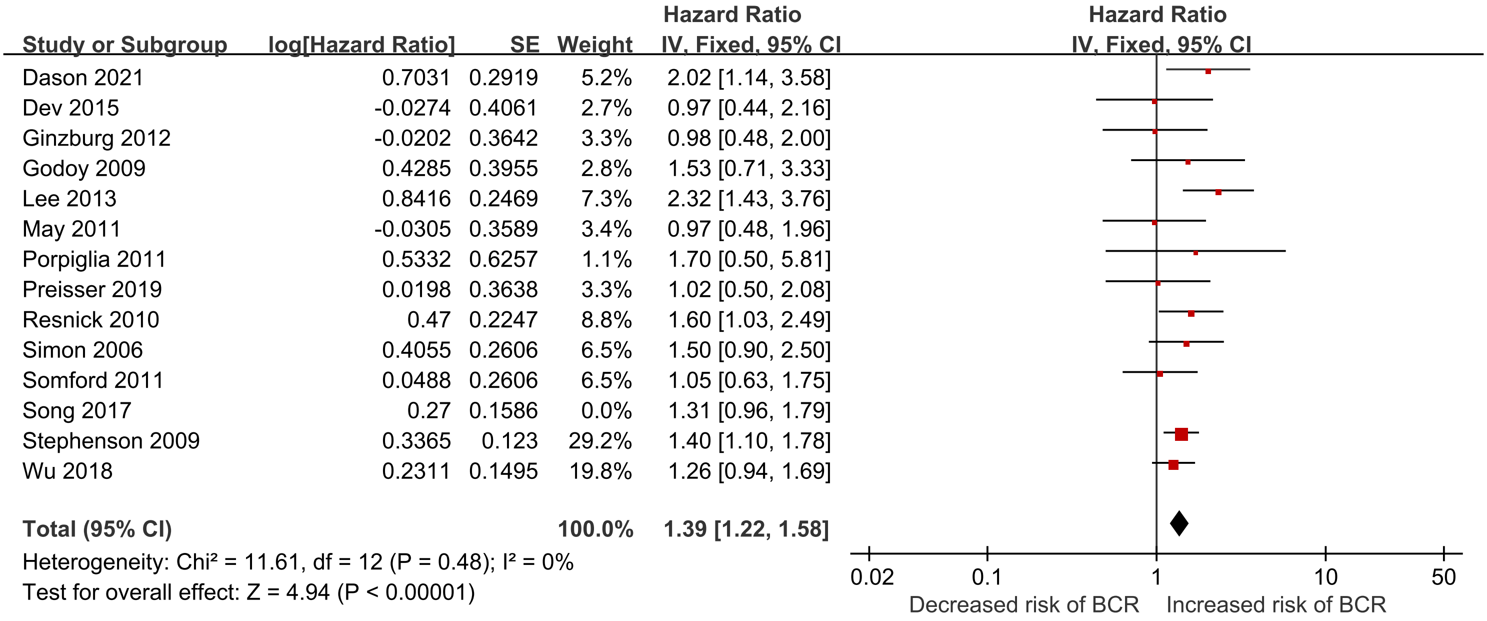

13. Forest plots of  studies excluded Stephenson's study evaluating the association between focality of PSM and BCR

| Study or Subgroup | log[Hazard Ratio] | SE | Weight | Hazard Ratio IV, Fixed, 95% CI |
|---|---|---|---|---|
| Dason 2021 | 0.7031 | 0.2919 | 5.9% | 2.02 [1.14, 3.58] |
| Dev 2015 | -0.0274 | 0.4061 | 3.0% | 0.97 [0.44, 2.16] |
| Ginzburg 2012 | -0.0202 | 0.3642 | 3.8% | 0.98 [0.48, 2.00] |
| Godoy 2009 | 0.4285 | 0.3955 | 3.2% | 1.53 [0.71, 3.33] |
| Lee 2013 | 0.8416 | 0.2469 | 8.2% | 2.32 [1.43, 3.76] |
| May 2011 | -0.0305 | 0.3589 | 3.9% | 0.97 [0.48, 1.96] |
| Porpiglia 2011 | 0.5332 | 0.6257 | 1.3% | 1.70 [0.50, 5.81] |
| Preisser 2019 | 0.0198 | 0.3638 | 3.8% | 1.02 [0.50, 2.08] |
| Resnick 2010 | 0.47 | 0.2247 | 9.9% | 1.60 [1.03, 2.49] |
| Simon 2006 | 0.4055 | 0.2606 | 7.4% | 1.50 [0.90, 2.50] |
| Somford 2011 | 0.0488 | 0.2606 | 7.4% | 1.05 [0.63, 1.75] |
| Song 2017 | 0.27 | 0.1586 | 19.9% | 1.31 [0.96, 1.79] |
| Stephenson 2009 | 0.3365 | 0.123 | 0.0% | 1.40 [1.10, 1.78] |
| Wu 2018 | 0.2311 | 0.1495 | 22.4% | 1.26 [0.94, 1.69] |
| **Total (95% CI)** | | | **100.0%** | **1.37 [1.19, 1.57]** |

Heterogeneity: Chi² = 11.70, df = 12 (P = 0.47); I² = 0%
Test for overall effect: Z = 4.45 (P < 0.00001)

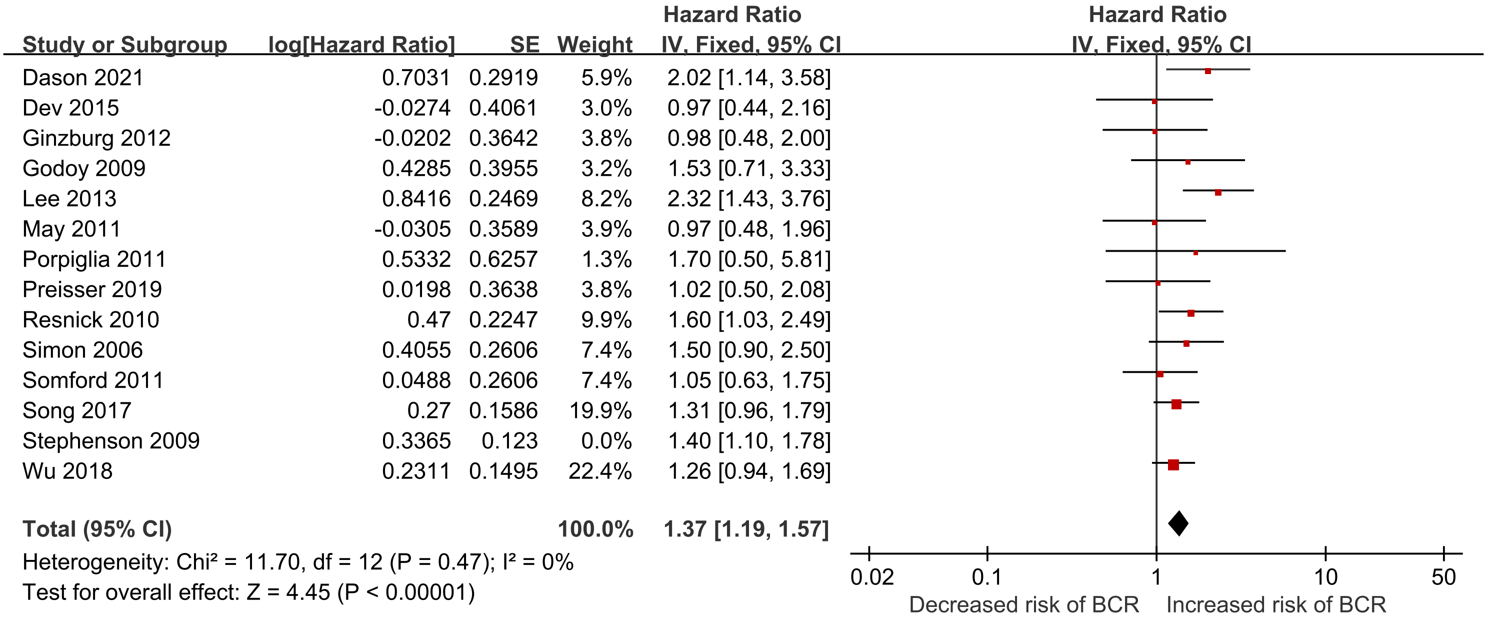

14. Forest plots of studies excluded Wu's study evaluating the association between focality of PSM and BCR

| Study or Subgroup | log[Hazard Ratio] | SE | Weight | Hazard Ratio IV, Fixed, 95% CI |
|---|---|---|---|---|
| Dason 2021 | 0.7031 | 0.2919 | 5.3% | 2.02 [1.14, 3.58] |
| Dev 2015 | -0.0274 | 0.4061 | 2.7% | 0.97 [0.44, 2.16] |
| Ginzburg 2012 | -0.0202 | 0.3642 | 3.4% | 0.98 [0.48, 2.00] |
| Godoy 2009 | 0.4285 | 0.3955 | 2.9% | 1.53 [0.71, 3.33] |
| Lee 2013 | 0.8416 | 0.2469 | 7.4% | 2.32 [1.43, 3.76] |
| May 2011 | -0.0305 | 0.3589 | 3.5% | 0.97 [0.48, 1.96] |
| Porpiglia 2011 | 0.5332 | 0.6257 | 1.2% | 1.70 [0.50, 5.81] |
| Preisser 2019 | 0.0198 | 0.3638 | 3.4% | 1.02 [0.50, 2.08] |
| Resnick 2010 | 0.47 | 0.2247 | 9.0% | 1.60 [1.03, 2.49] |
| Simon 2006 | 0.4055 | 0.2606 | 6.7% | 1.50 [0.90, 2.50] |
| Somford 2011 | 0.0488 | 0.2606 | 6.7% | 1.05 [0.63, 1.75] |
| Song 2017 | 0.27 | 0.1586 | 18.0% | 1.31 [0.96, 1.79] |
| Stephenson 2009 | 0.3365 | 0.123 | 29.9% | 1.40 [1.10, 1.78] |
| Wu 2018 | 0.2311 | 0.1495 | 0.0% | 1.26 [0.94, 1.69] |
| **Total (95% CI)** | | | **100.0%** | **1.40 [1.23, 1.60]** |

Heterogeneity: Chi² = 11.30, df = 12 (P = 0.50); I² = 0%
Test for overall effect: Z = 5.03 (P < 0.00001)

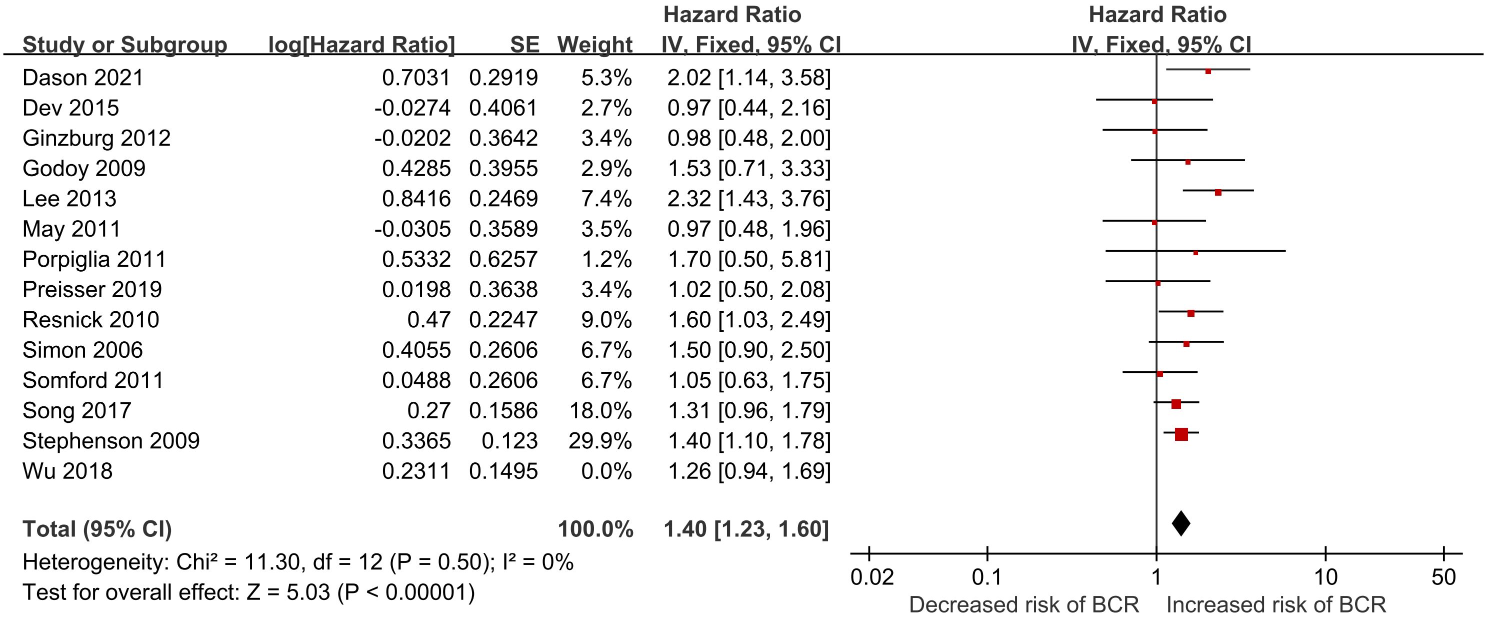

Supplement: S3 File — (PDF) [file pone.0301653.s006.pdf]
