# Supplementary material for: The impact of positive surgical margin parameters and pathological stage on biochemical recurrence after radical prostatectomy: A systematic review and meta-analysis
Source: PLoS One. 2024 Jul 11;19(7):e0301653. doi: 10.1371/journal.pone.0301653 (PMC11239040; doi:10.1371/journal.pone.0301653)

1. Forest plots of  studies excluded Ginzburg's study evaluating the association between pT3a/pT2 and BCR

| Study or Subgroup | log[Hazard Ratio] | SE | Weight | Hazard Ratio IV, Fixed, 95% CI |
|---|---|---|---|---|
| Ginzburg 2012 | 0.8329 | 0.2756 | 0.0% | 2.30 [1.34, 3.95] |
| Kordan 2009 | 0.9361 | 0.2009 | 56.8% | 2.55 [1.72, 3.78] |
| Oort 2010 | 0.1222 | 0.3582 | 17.9% | 1.13 [0.56, 2.28] |
| Porcaro 2019 | 0.4769 | 0.4654 | 10.6% | 1.61 [0.65, 4.01] |
| Sooriakumaran 2015 | 0.174 | 0.3938 | 14.8% | 1.19 [0.55, 2.57] |
| **Total (95% CI)** | | | **100.0%** | **1.88 [1.39, 2.52]** |

Heterogeneity: Chi² = 5.78, df = 3 (P = 0.12); I² = 48%

Test for overall effect: Z = 4.16 (P < 0.0001)

**Hazard Ratio**
**IV, Fixed, 95% CI**

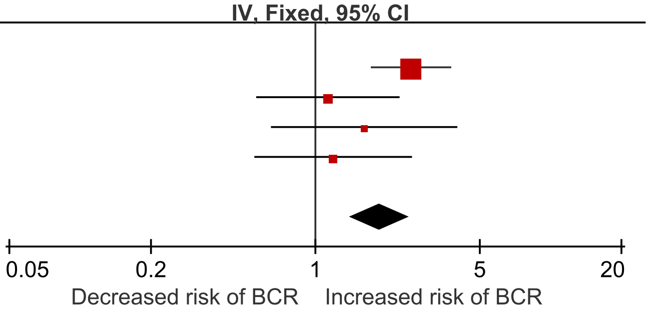

2. Forest plots of studies excluded Kordan's study evaluating the association between pT3a/pT2 and BCR

| Study or Subgroup | log[Hazard Ratio] | SE | Weight | Hazard Ratio IV, Fixed, 95% CI |
|---|---|---|---|---|
| Ginzburg 2012 | 0.8329 | 0.2756 | 41.1% | 2.30 [1.34, 3.95] |
| Kordan 2009 | 0.9361 | 0.2009 | 0.0% | 2.55 [1.72, 3.78] |
| Oort 2010 | 0.1222 | 0.3582 | 24.3% | 1.13 [0.56, 2.28] |
| Porcaro 2019 | 0.4769 | 0.4654 | 14.4% | 1.61 [0.65, 4.01] |
| Sooriakumaran 2015 | 0.174 | 0.3938 | 20.1% | 1.19 [0.55, 2.57] |
| **Total (95% CI)** | | | **100.0%** | **1.61 [1.14, 2.28]** |

Heterogeneity: Chi² = 3.24, df = 3 (P = 0.36); I² = 7%
Test for overall effect: Z = 2.69 (P = 0.007)

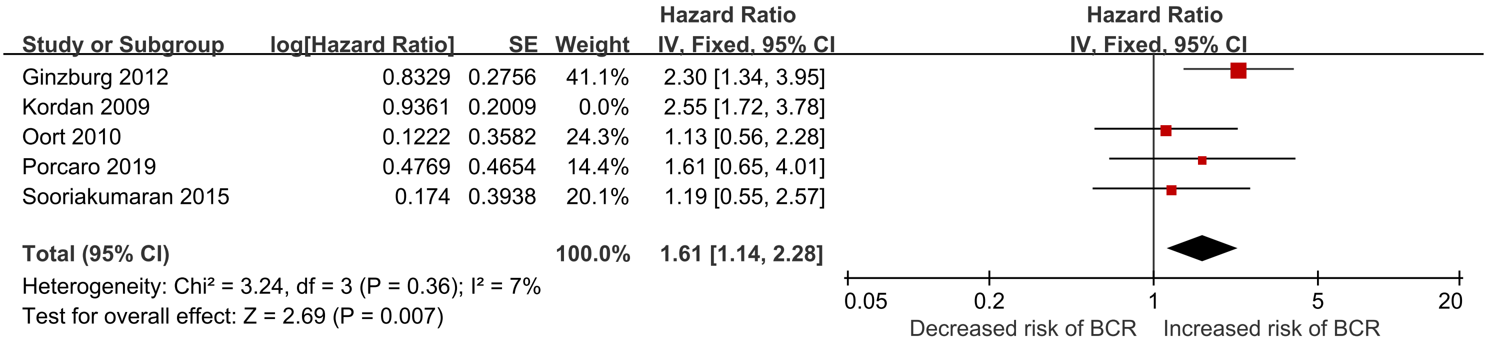

3. Forest plots of  studies excluded Oort's study evaluating the association between pT3a/pT2 and BCR

| Study or Subgroup | log[Hazard Ratio] | SE | Weight | Hazard Ratio IV, Fixed, 95% CI |
|---|---|---|---|---|
| Ginzburg 2012 | 0.8329 | 0.2756 | 26.9% | 2.30 [1.34, 3.95] |
| Kordan 2009 | 0.9361 | 0.2009 | 50.6% | 2.55 [1.72, 3.78] |
| Oort 2010 | 0.1222 | 0.3582 | 0.0% | 1.13 [0.56, 2.28] |
| Porcaro 2019 | 0.4769 | 0.4654 | 9.4% | 1.61 [0.65, 4.01] |
| Sooriakumaran 2015 | 0.174 | 0.3938 | 13.2% | 1.19 [0.55, 2.57] |
| **Total (95% CI)** | | | **100.0%** | **2.15 [1.62, 2.84]** |

Heterogeneity: Chi² = 3.42, df = 3 (P = 0.33); I² = 12%

Test for overall effect: Z = 5.35 (P < 0.00001)

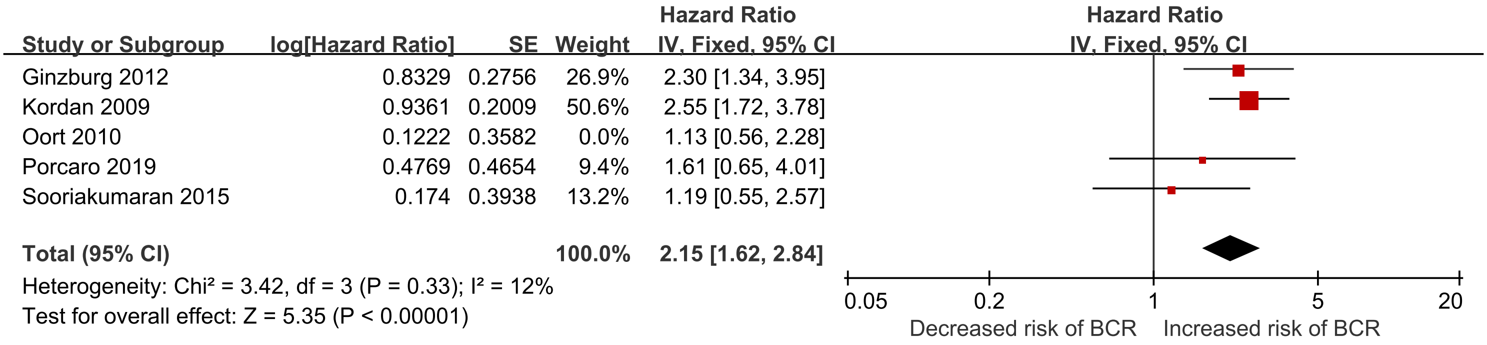

4. Forest plots of  studies excluded Porcaro's study evaluating the association between pT3a/pT2 and BCR

| Study or Subgroup | log[Hazard Ratio] | SE | Weight | Hazard Ratio IV, Fixed, 95% CI |
|---|---|---|---|---|
| Ginzburg 2012 | 0.8329 | 0.2756 | 25.2% | 2.30 [1.34, 3.95] |
| Kordan 2009 | 0.9361 | 0.2009 | 47.5% | 2.55 [1.72, 3.78] |
| Oort 2010 | 0.1222 | 0.3582 | 14.9% | 1.13 [0.56, 2.28] |
| Porcaro 2019 | 0.4769 | 0.4654 | 0.0% | 1.61 [0.65, 4.01] |
| Sooriakumaran 2015 | 0.174 | 0.3938 | 12.4% | 1.19 [0.55, 2.57] |
| **Total (95% CI)** | | | **100.0%** | **2.00 [1.53, 2.63]** |

Heterogeneity: Chi² = 6.00, df = 3 (P = 0.11); I² = 50%

Test for overall effect: Z = 5.02 (P < 0.00001)

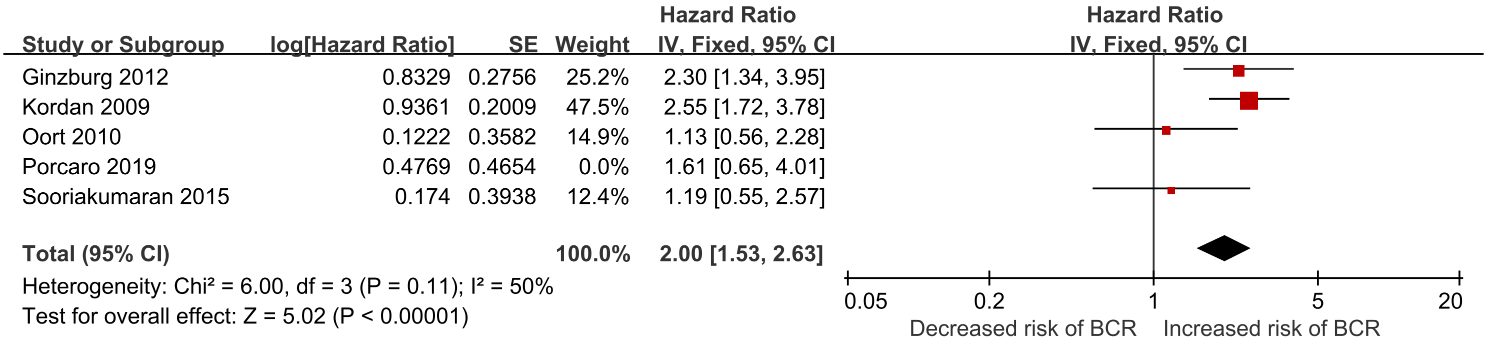

5. Forest plots of  studies excluded Soorialumaran's study evaluating the association between pT3a/pT2 and BCR

| Study or Subgroup | log[Hazard Ratio] | SE | Weight | Hazard Ratio IV, Fixed, 95% CI |
|---|---|---|---|---|
| Ginzburg 2012 | 0.8329 | 0.2756 | 26.1% | 2.30 [1.34, 3.95] |
| Kordan 2009 | 0.9361 | 0.2009 | 49.2% | 2.55 [1.72, 3.78] |
| Oort 2010 | 0.1222 | 0.3582 | 15.5% | 1.13 [0.56, 2.28] |
| Porcaro 2019 | 0.4769 | 0.4654 | 9.2% | 1.61 [0.65, 4.01] |
| Sooriakumaran 2015 | 0.174 | 0.3938 | 0.0% | 1.19 [0.55, 2.57] |
| **Total (95% CI)** | | | **100.0%** | **2.10 [1.59, 2.77]** |

Heterogeneity: Chi² = 4.36, df = 3 (P = 0.23); I² = 31%
Test for overall effect: Z = 5.26 (P < 0.00001)

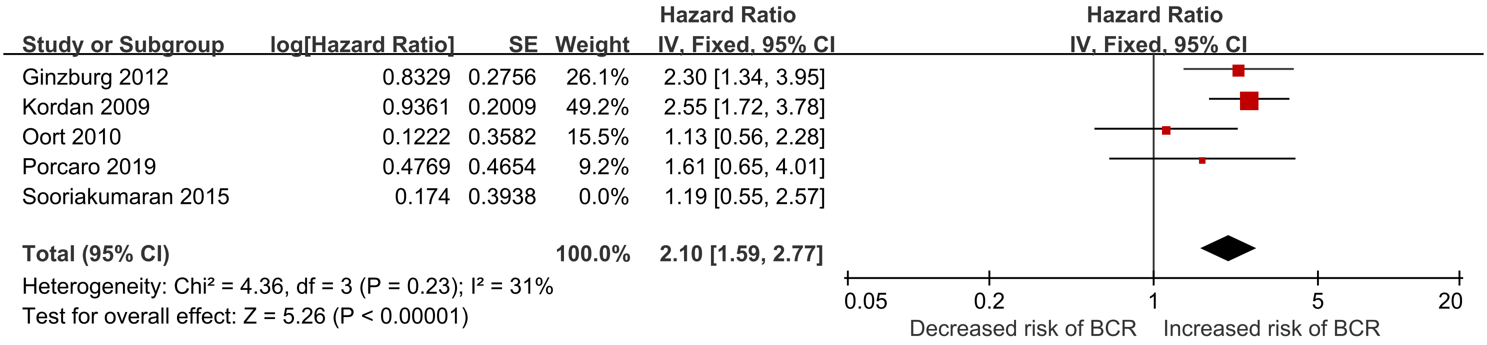

Supplement: S4 File — (PDF) [file pone.0301653.s007.pdf]
