# Supplementary material for: The impact of positive surgical margin parameters and pathological stage on biochemical recurrence after radical prostatectomy: A systematic review and meta-analysis
Source: PLoS One. 2024 Jul 11;19(7):e0301653. doi: 10.1371/journal.pone.0301653 (PMC11239040; doi:10.1371/journal.pone.0301653)
Supplement: S3 Text — (DOCX) [file pone.0301653.s011.docx]

**S3 Text. Search strategy.**

**Cochrane Library**

**Prostatic Neoplasms**

Synonyms: Prostate Neoplasms; Prostatic Neoplasm; Neoplasm, Prostatic; Neoplasm, Prostate; Prostate Neoplasm; Neoplasms, Prostatic; Neoplasms, Prostate; Prostatic Cancers; Cancers, Prostatic; Cancer of the Prostate; Prostate Cancers; Cancer of Prostate; Cancer, Prostate; Prostate Cancer; Cancers, Prostate; Cancer, Prostatic; Prostatic Cancer

**Surgical Margin**

Synonyms: Margin, Resection; Surgical Margins; Margins, Surgical; Margins, Resection; Surgical Margin; Resection Margin; Margin, Surgical; Excision Margins; Excision Margin; Resection Margins; Positive Surgical Margins; Surgical Margin, Positive; Positive Surgical Margin; Surgical Margins, Positive; Negative Surgical Margins; Surgical Margin, Negative; Margin, Tumor-Free; Tumor Free Margins; Negative Surgical Margin; Surgical Margins, Negative; Tumor-Free Margins; Margins, Tumor-Free; Tumor-Free Margin

**Prostatectomy**

Synonyms: Prostatectomies, Retropubic; Prostatectomy, Retropubic; Retropubic Prostatectomies; Retropubic Prostatectomy; Suprapubic Prostatectomies; Suprapubic Prostatectomy; Prostatectomies, Suprapubic; Prostatectomy, Suprapubic; Prostatectomies

**PubMed**

**Prostate Cancer**

Prostate Neoplasms; Neoplasms, Prostate; Neoplasm, Prostate; Prostate Neoplasm; Neoplasms, Prostatic; Neoplasm, Prostatic; Prostatic Neoplasm; Prostate Cancer; Cancer, Prostate; Cancers, Prostate; Prostate Cancers; Cancer of the Prostate; Prostatic Cancer; Cancer, Prostatic; Cancers, Prostatic; Prostatic Cancers; Cancer of Prostate

**Positive margin**

Excision Margin; Excision Margins; Resection Margin; Margin, Resection; Margins, Resection; Resection Margins; Surgical Margins; Margin, Surgical; Margins, Surgical; Surgical Margin; Positive Surgical Margins; Positive Surgical Margin; Surgical Margin, Positive; Surgical Margins, Positive; Negative Surgical Margins; Negative Surgical Margin; Surgical Margin, Negative; Surgical Margins, Negative; Tumor-Free Margins; Margin, Tumor-Free; Margins, Tumor-Free; Tumor Free Margins; Tumor-Free Margin

# **Prostatectomy**

Prostatectomies; Prostatectomy, Suprapubic; Prostatectomies, Suprapubic; Suprapubic Prostatectomies; Suprapubic Prostatectomy; Prostatectomy, Retropubic; Prostatectomies, Retropubic; Retropubic Prostatectomies; Retropubic Prostatectomy

**Embase**

**prostate cancer**

Synonyms

cancer, prostate; malignant prostate tumor; malignant prostate tumour; malignant prostatic tumor; malignant prostatic tumour; prostate gland cancer; prostate malignancy; prostate malignant neoplasm; prostate malignant tumor; prostate malignant tumour; prostatic cancer; prostatic malignancy

**Surgical Margin**

Synonyms

excision margin; excision margins; margin of excision; margins of excision; normal tissue margin; normal tissue margins; resection margin; resection margins; surgical margin of excision; surgical margins; surgical margins of excision; tumor-free margin; tumor-free margins

**Prostatectomy**

Synonyms

prostate adenectomy; prostate resection; prostatic adenectomy; radical prostatectomy; total prostatectomy
