# Supplementary material for: The impact of positive surgical margin parameters and pathological stage on biochemical recurrence after radical prostatectomy: A systematic review and meta-analysis
Source: PLoS One. 2024 Jul 11;19(7):e0301653. doi: 10.1371/journal.pone.0301653 (PMC11239040; doi:10.1371/journal.pone.0301653)
Supplement: S4 Text — (DOCX) [file pone.0301653.s012.docx]

| **1. Did the research questions and inclusion criteria for the review include the components of PICO?**   \| For Yes: Optional (recommended)  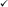 Timeframe for follow-up 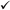Yes  □ No  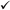 Population  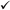 Intervention  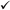 Comparator group  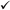 Outcome \| \| --- \|   **2. Did the report of the review contain an explicit statement that the review methods were**  **established prior to the conduct of the review and did the report justify any significant deviations from the protocol?**   \| For Yes:  As for partial yes, plus the protocol should be registered and should also have specified:  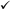 Yes  □ Partial Yes □ No  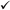  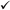  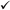  a meta-analysis/synthesis plan, if appropriate, *and*  a plan for investigating causes of heterogeneity  For Partial Yes:  The authors state that they had a written protocol or guide that included ALL the following:  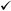 review question(s)  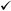 asearch strategy  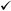 inclusion/exclusion criteria  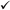 a risk of bias assessment  justification for any deviations from the protocol \| \| --- \|   **3. Did the review authors explain their selection of the study designs for inclusion in the review?**   \| \| For Yes, the review should satisfy ONE of the following: \|  \| \| --- \| --- \| \| □ *Explanation for* including only RCTs  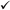 OR *Explanation for* including only NRSI  □ OR *Explanation for* including both RCTs and NRSI \| 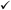 Yes □ No \| \| \| --- \| --- \| --- \| --- \| --- \|   **4. Did the review authors use a comprehensive literature search strategy?**   \| For Yes, should also have (all the following):  For Partial Yes (all the following):  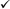 searched at least 2 databases (relevant to research question)  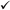 provided keyword and/or search strategy  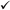 justified publication restrictions (e.g. language)  □ 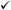  □  Yes  Partial Yes No  □ searched the reference lists / bibliographies of included  studies  □ searched trial/study registries  □ included/consulted content experts in the field  □ where relevant, searched for grey literature  □ conducted search within 24 months of completion of the review \| \| --- \|   **5. Did the review authors perform study selection in duplicate?**   \| For Yes, either ONE of the following:  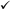 Yes □ No  □ at least two reviewers independently agreed on selection of eligible studies and achieved consensus on which studies to include  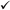 OR two reviewers selected a sample of eligible studies and achieved good agreement (at least 80 percent), with the remainder selected by one  reviewer. \| \| --- \| |
| --- | --- | --- | --- | --- | --- | --- | --- | --- | --- |

| **6. Did the review authors perform data extraction in duplicate?**   \| For Yes, either ONE of the following:  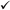 Yes □ No  □ at least two reviewers achieved consensus on which data to extract from included studies  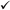 OR two reviewers extracted data from a sample of eligible studies and achieved good agreement (at least 80 percent), with the remainder  extracted by one reviewer. \| \| --- \|   **7. Did the review authors provide a list of excluded studies and justify the exclusions?**   \| For Partial Yes:  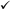 Yes  □ Partial Yes □ No  □ provided a list of all potentially relevant studies that were read in full-text form but excluded from the review  For Yes, must also have:  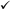 Justified the exclusion from the review of each potentially relevant study \| \| --- \|   **8. Did the review authors describe the included studies in adequate detail?**   \| For Yes, should also have ALL the following:  For Partial Yes (ALL the following):   \| □ □ □ □ □ \| described described described described described \| populations interventions comparators outcomes  research designs \| \| --- \| --- \| --- \|   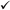 Yes  □ Partial Yes □ No  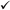 described population in detail  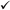 described intervention in  detail (including doses where relevant)  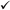 described comparator in detail (including doses where  relevant)  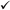 described study’s setting  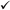 timeframe for follow-up \| \| --- \| --- \| --- \| --- \|   **9. Did the review authors use a satisfactory technique for assessing the risk of bias (RoB) in**  **individual studies that were included in the review?**   \| **RCTs**  □ Yes  □ Partial Yes □ No  □ Includes only NRSI  For Partial Yes, must have assessed RoB from  For Yes, must also have assessed RoB from:  □ allocation sequence that was not truly random, *and*  □ selection of the reported result from among multiple  measurements or analyses of a specified outcome  □ unconcealed allocation, *and*  □ lack of blinding of patients and assessors when assessing  outcomes (unnecessary for  objective outcomes such as all- cause mortality) \| \| --- \| \| **NRSI**  For Partial Yes, must have assessed RoB:  For Yes, must also have assessed RoB:  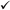 methods used to ascertain  exposures and outcomes, *and*  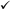 selection of the reported result from among multiple  measurements or analyses of a specified outcome  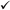 Yes  □ Partial Yes □ No  □ Includes only RCTs  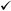 from confounding, *and*  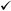 from selection bias \| |
| --- | --- | --- | --- | --- | --- | --- | --- | --- |
| 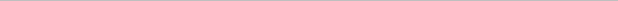**10. Did the review authors report on the sources of funding for the studies included in the review?**  For Yes  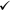 Must have reported on the sources of funding for individual studies included in the review. Note: Reporting that the reviewers looked for this information but it was not reported by study authors also qualifies  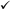 Yes □ No |

| **11. If meta-analysis was performed did the review authors use appropriate methods for statistical**  **combination of results?**   \| **RCTs** \|  \|  \| \| --- \| --- \| --- \| \| For Yes: \|  \|  \| \| □ The authors justified combining the data in a meta-analysis \| □ \| Yes \| \| □ AND they used an appropriate weighted technique to combine \| □ \| No \| \| study results and adjusted for heterogeneity if present. \| □ \| No meta-analysis \| \| □ AND investigated the causes of any heterogeneity \|  \| conducted \| \| **For NRSI** \|  \|  \| \| For Yes: \|  \|  \| \| 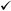 The authors justified combining the data in a meta-analysis \| 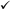 \| Yes \| \| 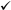 AND they used an appropriate weighted technique to combine \| □ \| No \| \| study results, adjusting for heterogeneity if present \| □ \| No meta-analysis \| \| 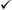 AND they statistically combined effect estimates from NRSI that were adjusted for confounding, rather than combining raw data,  or justified combining raw data when adjusted effect estimates were not available \|  \| conducted \| \| 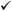 AND they reported separate summary estimates for RCTs and NRSI separately when both were included in the review \|  \|  \|   **12. If meta-analysis was performed, did the review authors assess the potential impact of RoB in**  **individual studies on the results of the meta-analysis or other evidence synthesis?**   \| For Yes:  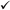 Yes □ No  □ No meta-analysis conducted  □ included only low risk of bias RCTs  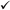 OR, if the pooled estimate was based on RCTs and/or NRSI at variable RoB, the authors performed analyses to investigate possible impact of RoB on summary estimates of effect. \| \| --- \|   **13. Did the review authors account for RoB in individual studies when interpreting/ discussing the**  **results of the review?**   \| \| For Yes: \|  \| \| --- \| --- \| \| □ included only low risk of bias RCTs  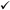 OR, if RCTs with moderate or high RoB, or NRSI were included the review provided a discussion of the likely impact of RoB on the results \| 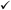 Yes □ No \| \| \| --- \| --- \| --- \| --- \| --- \|   **14. Did the review authors provide a satisfactory explanation for, and discussion of, any**  **heterogeneity observed in the results of the review?**   \| For Yes:  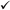 Yes □ No  □ There was no significant heterogeneity in the results  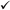 OR if heterogeneity was present the authors performed an investigation of sources of any heterogeneity in the results and discussed the impact of this on the results of the review \| \| --- \|   **15. If they performed quantitative synthesis did the review authors carry out an adequate**  **investigation of publication bias (small study bias) and discuss its likely impact on the results of the review?**   \| For Yes:  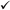 performed graphical or statistical tests for publication bias and discussed the likelihood and magnitude of impact of publication bias  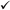 Yes □ No  □ No meta-analysis conducted \| \| --- \| |
| --- | --- | --- | --- | --- | --- | --- | --- | --- | --- | --- | --- | --- | --- | --- | --- | --- | --- | --- | --- | --- | --- | --- | --- | --- | --- | --- | --- | --- | --- | --- | --- | --- | --- | --- | --- | --- | --- | --- | --- | --- | --- | --- | --- | --- | --- | --- | --- |

| **16. Did the review authors report any potential sources of conflict of interest, including any funding**  **they received for conducting the review?** | | |
| --- | --- | --- |
| For Yes:  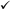 The authors reported no competing interests OR  □ The authors described their funding sources and how they managed | 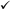 Yes □ No |  |
| potential conflicts of interest | |  |

**To cite this tool:** Shea BJ, Reeves BC, Wells G, Thuku M, Hamel C, Moran J, Moher D, Tugwell P, Welch V, Kristjansson E, Henry DA. AMSTAR 2: a critical appraisal tool for systematic reviews that include randomised or non-randomised studies of healthcare interventions, or both. BMJ. 2017 Sep 21;358:j4008.
